# Structural basis of GM-CSF and IL-2 sequestration by the viral decoy receptor GIF

**DOI:** 10.1038/ncomms13228

**Published:** 2016-11-07

**Authors:** Jan Felix, Eaazhisai Kandiah, Steven De Munck, Yehudi Bloch, Gydo C.P. van Zundert, Kris Pauwels, Ann Dansercoer, Katka Novanska, Randy J. Read, Alexandre M.J.J. Bonvin, Bjorn Vergauwen, Kenneth Verstraete, Irina Gutsche, Savvas N. Savvides

**Affiliations:** 1 Laboratory for Protein Biochemistry and Biomolecular Engineering, Department of Biochemistry and Microbiology, Ghent University, Technologiepark 927, 9052 Ghent, Belgium; 2 VIB Inflammation Research Center, Technologiepark 927, 9052 Ghent, Belgium; 3 University Grenoble Alpes, CNRS, CEA, IBS, F-38044 Grenoble, France; 4 Bijvoet Center for Biomolecular Research, Faculty of Science, Utrecht University, 3584CH Utrecht, The Netherlands; 5 VIB Structural Biology Research Center, Vrije Universiteit Brussel, Pleinlaan 2, 1040 Brussels, Belgium; 6 Masaryk University & CEITEC, 62500 Brno, Czech Republic; 7 Department of Haematology, University of Cambridge School of Clinical Medicine, Cambridge Institute for Medical Research, Wellcome Trust/MRC Building, Cambridge Biomedical Campus Box 139, Cambridge CB2 0XY, UK

## Abstract

Subversion of the host immune system by viruses is often mediated by molecular decoys that sequester host proteins pivotal to mounting effective immune responses. The widespread mammalian pathogen parapox Orf virus deploys GIF, a member of the poxvirus immune evasion superfamily, to antagonize GM-CSF (granulocyte macrophage colony-stimulating factor) and IL-2 (interleukin-2), two pleiotropic cytokines of the mammalian immune system. However, structural and mechanistic insights into the unprecedented functional duality of GIF have remained elusive. Here we reveal that GIF employs a dimeric binding platform that sequesters two copies of its target cytokines with high affinity and slow dissociation kinetics to yield distinct complexes featuring mutually exclusive interaction footprints. We illustrate how GIF serves as a competitive decoy receptor by leveraging binding hotspots underlying the cognate receptor interactions of GM-CSF and IL-2, without sharing any structural similarity with the cytokine receptors. Our findings contribute to the tracing of novel molecular mimicry mechanisms employed by pathogenic viruses.

## Abstract (web summary)

Viruses often subvert the host immune system using molecular decoys to prevent an effective immune response. Here, the authors examine the structural details of the viral decoy receptor GIF and its antagnosim of GM-CSF and IL-2.

## Graphical abstract

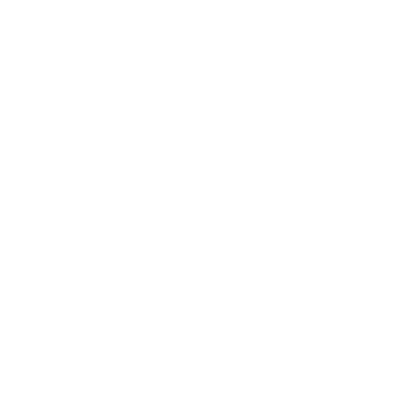

Throughout evolution, mammalian viruses co-evolved with their hosts and developed numerous immunomodulatory strategies aimed at countering and evading antiviral responses by the host immune system. Among the many different immune evasion tactics deployed by viruses, are the use of antigenic variation, latency, interference with antigen presentation, inhibition of apoptosis of viral infected cells and viral mimicry^1^,^2^. The latter is mainly deployed by large DNA viruses of the pox and herpes family, which encode viral homologues of host cytokines, chemokines and their cognate receptors as primary molecular detractors of the host immune response^3^,^4^,^5^.

The orf virus, a 140 kb double-stranded DNA (dsDNA) virus from the parapoxfamily infects ruminants and humans worldwide causing contagious ecthyma, also known as orf, a highly spreadable skin condition characterized by severe lesions and ulcerations^6^,^7^,^8^,^9^. As such, the socioeconomic footprint of orf is estimated to be very large placing orf in the top 20 of the most impactful diseases in developing countries or poor communities with a heavy economic dependence on animal farming and agrarian activities^10^,^11^. Infection by the Orf virus through damaged skin elicits a severe and acute immune response characterized by local accumulation of T cells, B cells, dermal dendritic cells and neutrophils^12^,^13^. Although the Orf virus does not inflict systemic infections and orf is generally not fatal, it can lead to high mortality rates in young animals and children when ulcerations prohibit food intake or can drastically affect ovine flock sizes when lesions would hinder reproduction^8^,^14^.

The Orf virus subverts the immune response by secreting virulence proteins that inhibit or mimic key anti-inflammatory host effector molecules^15^, including a chemokine-binding protein (CKBP)^16^, an Orf virus homologue of interleukin-10 (orfIL-10)^17^ and vascular endothelial growth factor (VEGF-E)^18^, an interferon resistance protein (OVIFNR) resembling the vaccinia virus E3L gene^19^ and a secreted dual inhibitor of both granulocyte macrophage colony-stimulating factor (GM-CSF) and interleukin-2 (IL-2), termed GM-CSF/IL-2 inhibition factor (GIF)^20^.

The latter, GIF, is arguably one of the most intriguing viral proteins known because it targets two distinct host proteins pivotal to the host's ability to mount an effective immune response. For instance, GM-CSF is involved in the survival and activation of macrophages, neutrophils and eosinophils, the maturation of dendritic cells and differentiation of invariant natural killer T cells^21^, while IL-2 is crucial for the maintenance of regulatory T cells, and the proliferation of B cells, memory T cells and NK cells^22^,^23^. GIF is distantly related to type-II CKBP (CKBP-II), also known as the ‘vCCI' or ‘orthopox 35kDa major secreted virus' family^24^,^25^,^26^, and displays restrictive cross-species specificity since it is able to bind ovine GM-CSF and IL-2, but not their human or murine orthologues^20^. However, the structural and mechanistic basis for the remarkable functional duality displayed by GIF has remained uncharacterized.

In this study, we employ an integrative structural biology approach in an effort to elucidate the structural and mechanistic principles of how a single viral protein can sequester two different host proteins. Via a series of cross-validated structural snapshots including the crystal structure of GIF in complex with GM-CSF, two-dimensional (2D) classifications of GIF:GM-CSF and GIF:IL-2 complexes by negative-stain electron microscopy, the ensuing three-dimensional (3D) reconstruction of the GIF:IL-2 complex, and modeling of small-angle X-ray scattering (SAXS) data in conjunction with multi-angle laser light scattering (MALLS) measurements, we show that GIF adopts an obligate dimeric structure that can bind two molecules of its target cytokines. We further show via structure-based mutagenesis in combination with interaction studies that GIF does so with high affinity using a mutually exclusive interaction site for both cytokines and plays into binding principles utilized by the cognate receptors of GM-CSF and IL-2. Together, our findings help to unmask the molecular virtuosity gained during viral protein evolution towards new protein ligand specificities and the molecular mechanisms developed by viruses to subvert the host immune response.

## Results

### Crystal structure of the GIF:GM-CSF complex

To obtain structural insights at high resolution into the functional duality of GIF as a cytokine-binding platform we sought to determine the structure of GIF in complex with its target cytokines. Large-scale production of recombinant GIF by transient expression in HEK293T cells and subsequent purification by immobilized-metal affinity chromatography (IMAC) and size-exclusion chromatography (SEC) initially led to prohibitory low amounts of purified GIF protein and uncovered the tendency for recombinant GIF to aggregate in standard purification buffers. However, addition of a molar excess of either recombinant ovine GM-CSF or IL-2 to culture medium containing secreted recombinant GIF enabled *in situ* reconstitution of GIF:GM-CSF and GIF:IL-2 complexes that could be purified to homogeneity for further biophysical and structural studies. Indeed, crystallization trials and subsequent crystal optimization efforts yielded crystals of the GIF:GM-CSF complex that enabled determination of the crystal structure to 2.8 Å resolution (Table 1) allowing us to obtain for the first time structural snapshots of GIF and its complex with GM-CSF.

**Figure 1:**
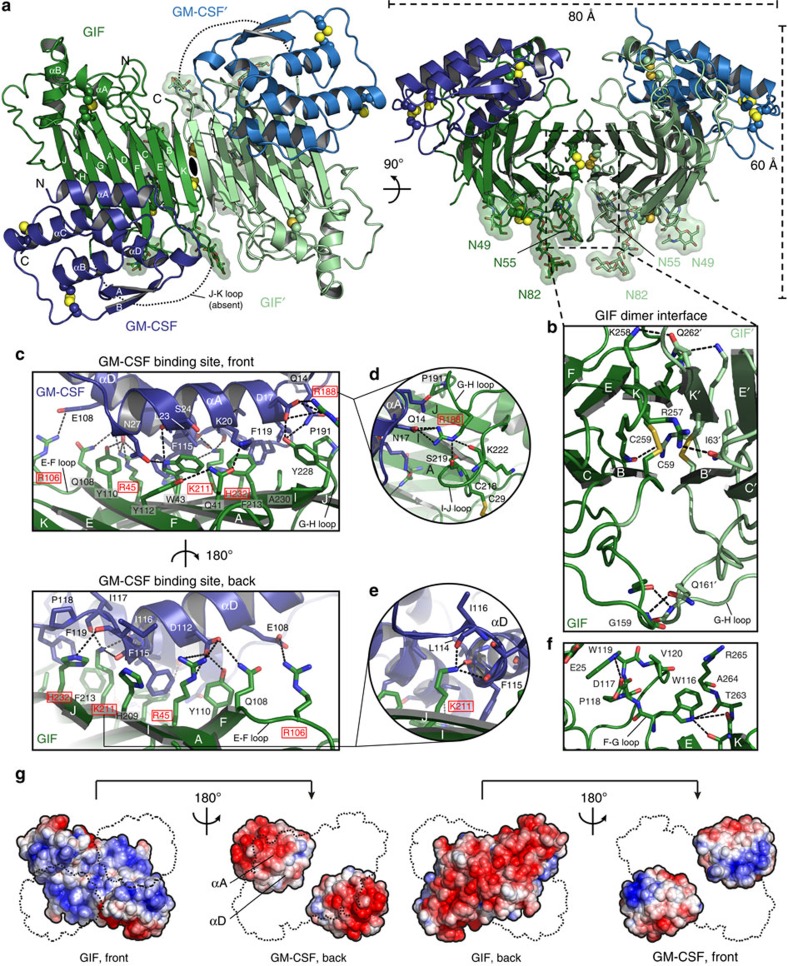
Crystal structure of the GIF:GM-CSF complex. (**a**) Dimeric GIF (pale green/dark green) is bound by two copies of ovine GM-CSF (blue/dark blue). Disulfides are shown as yellow spheres and N-linked glycans are depicted as sticks with transparent surface. The missing loop connecting β-strands βJ and βK is represented as a dashed line. The two-fold non-crystallographic symmetry (NCS) axis present at the center of the GIF dimer interface is indicated as a black oval. (**b**) Inset showing the GIF dimerization interface. Two GIF monomers are coloured in pale and dark green, disulfides are shown as yellow sticks and polar interactions are shown as dashed lines. (**c**) Inset of the GM-CSF (blue) binding epitope on GIF (green). Interacting residues are shown as sticks, and hydrogen bonds/salt-bridges are shown as dashed lines. Interface residues for which mutation data is available are labeled in red (see also Fig. 6; Supplementary Fig. 5; Supplementary Table 5). (**c**,**d**) Close-up view of interactions made by GIF residues R188 and K211, respectively. (**e**) Close-up view of interactions made by GIF residues R188 and K211, respectively. (**f**) Inset displaying the WSXWS-like motif (WDPWV) present in the F-G loop of GIF, which is involved in stabilizing the final three C-terminal residues of β-strand K. (**g**) Solvent accessible surface potential representation of the GIF:GM-CSF complex and individual subunits. The cytokine binding side of GIF is characterized by a basic surface potential complementary to the acidic patch formed by helices αA and αD of GM-CSF. Visualizations were generated using the PDB2PQR^74^ and APBS^75^ software.

**Figure 2:**
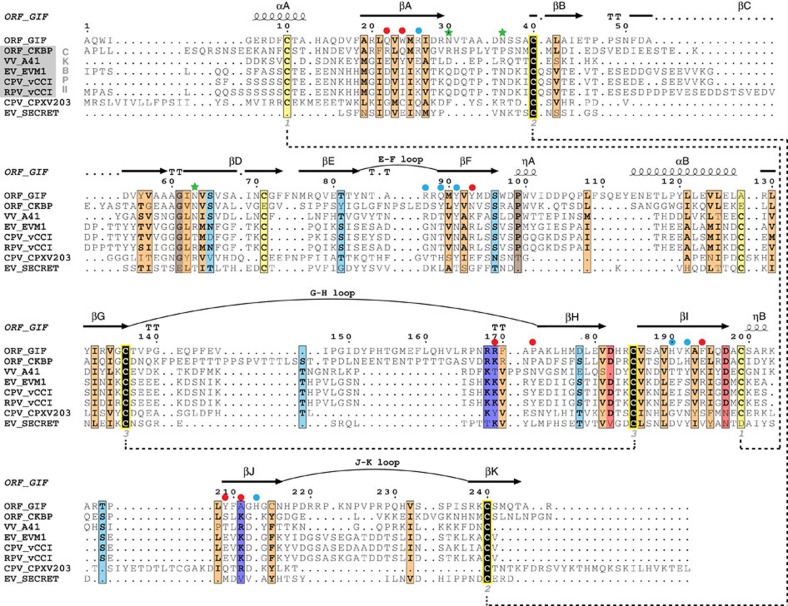
Sequence alignment of Orf GIF and members of the Poxviral Immune Evasion (PIE) family. Structural alignment performed by Expresso^76^ and visualized by ESPript^77^. Used sequences include Orf virus (ORF) CKBP, vaccinia virus (VV) A41, ectromelia virus (EV) vCCI (EVM1), cowpox virus (CPV) VCCI, rabbitpox virus (RPV) vCCI, cowpox virus (CPV) CPXV203 and ectromelia virus (EV) SECRET domain of CrmD. Conserved negatively and positively charged residues are shown in red and blue respectively, conserved polar residues are shown in light blue and conserved hydrophobic residues are shown in orange. Fully conserved residues are coloured black, with a yellow outline for cysteine. Conserved disulfide bonds are shown as connecting dashed lines. Secondary structure elements in GIF are annotated above the corresponding sequences. Residues of GIF involved in interactions with GM-CSF are shown as circles (red: interaction with helix αA, blue: interaction with helix αD of GM-CSF), whereas N-linked glycosylation sites in GIF are marked with a green star.

Our structural analyses reveal that GIF is an obligatory dimer, consisting of two GIF subunits with an 11-strand β-sandwich topology stabilized by three intramolecular disulfide bonds. Two GIF protomers interact via β-strand complementation mediated by strand βB and the C-terminal strand βK, resulting in an extended and concave β-sandwich platform (Fig. 1a). The ensuing dimeric GIF assembly is decorated by long loops and two short α-helices at the ends of each GIF protomer, with a dense cluster of N-linked glycans on the bottom side (Fig. 1a). The GIF dimer interface is additionally stabilized by two sets of interactions, one cluster centered around the intersheet disulfide bridge between Cys59 on strand βB and Cys59 on βK on either side of the twofold axis, and a second set mediated by the tips of opposing G-H loops (Fig. 1b; Supplementary Table 1). Interestingly, predictions of structural disorder identify the C-terminal 30 residues of GIF as intrinsically disordered. It is now clear that the second half of that sequence is essential for constructing the dimeric core structure of GIF, while the first half in the JK loop could not be modeled and might indeed be intrinsically disordered. The β-sandwich core of GIF is very similar to the fold representing poxviral CKBPs of the poxviral immune evasion (PIE) superfamily (Fig. 2) consistent with prior predictions and structural annotations^27^,^28^,^29^,^30^,^31^.

The GIF:GM-CSF complex features two molecules of GM-CSF at equivalent interaction epitopes at the poles of the dimeric β-sandwich platform (Fig. 1a). GM-CSF employs the helix-bundle face defined by helices αA and αD to interact with a rather broad surface on GIF contributed by strands βF, βA, βI and βJ burying ∼1,750 Å^2^ of solvent-accessible surface area (Fig. 1c; Supplementary Table 2). The interaction interface displays striking electrostatic complementarity with GIF contributing a highly basic surface flanking an island of aromatic residues, while GM-CSF projects a conspicuously acidic face of equal extent (Fig. 1g; Supplementary Table 2). In contrast, the side of GIF not involved in binding to GM-CSF displays opposite electrostatic properties in that is highly acidic (Fig. 1g) and heavily glycosylated (Fig. 1a).

Given the extensive interactions between GM-CSF and GIF, we wondered whether GM-CSF undergoes any conformational changes upon binding to GIF. To enable such comparisons, we determined the crystal structure of ovine GM-CSF (Table 1). Structural superposition of the bound and unbound forms of GM-CSF reveals that the helical bundle core of GM-CSF only undergoes rather subtle main-chain and side-chain adjustments, most notably Lys20 and Asn27 on helix αA, which tilt downwards to interact with Gln41 and Trp43 on βA of GIF, respectively (Supplementary Fig. 1). Furthermore, Glu108 on helix αD reaches out to Arg106 on the E-F loop of GIF, whereas Phe115 bends inwards to make place for His209 on βI of GIF. We further note that in one of the two-bound GM-CSF molecules, the N-terminal loop preceding helix αA at Gln14 is disordered, whereas in the second copy of GM-CSF it swings inwards by almost 180° to align roughly parallel to helix αA and interact with Phe189 and Tyr228 on GIF (Supplementary Fig. 1; Supplementary Table 2).

### GIF–cytokine complexes are structurally distinct

**Figure 3:**
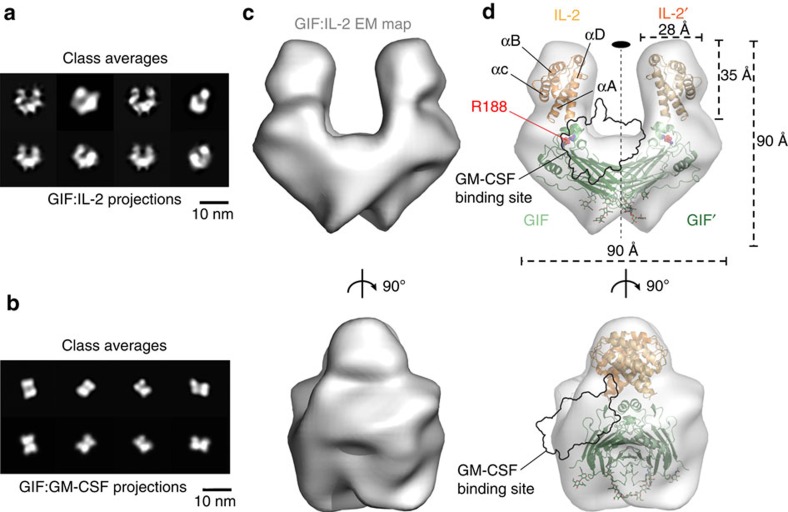
Structural analysis of GIF:IL-2 and GIF:GM-CSF complexes by negative-stain EM. (**a**) Gallery of representative *ab initio* class averages of the GIF:IL-2 complex and projections of the final 3D reconstruction under similar orientations. (**b**) *Ab initio* class averages of the GIF:GM-CSF complex and projections of the GIF:GM-CSF crystal structure under similar orientations. (**c**) Front and side views of a 3D reconstructed EM map for the GIF:IL-2 complex. The 3D volume displays a characteristic horseshoe shape. (**d**) Fit of the crystal structure of dimeric GIF and a homology model of ovine IL-2 in the corresponding EM map. The resulting model displays a horseshoe shaped complex formed by a GIF dimer bound by two copies of IL-2 (GIF dimer: green/dark green, IL-2: orange/dark orange) and displays a two-fold symmetry axis centered at the GIF dimer interface, indicated as a black oval.

Next we sought to obtain comparative structural information on GIF:GM-CSF and GIF:IL-2 complexes by negative-stain electron microscopy (EM) using purified complexes following co-expression of GIF with each of its target cytokines in HEK293T cells (Fig. 3a,b; Supplementary Fig. 2). The ensuing 2D class averages for the two complexes revealed particles with apparent twofold symmetry, albeit with striking differences. On one hand, the 2D class averages for the GIF:GM-CSF complex (Fig. 3b) agreed very well with the crystal structure for the GIF:GM-CSF complex (Fig. 1a). On the other hand, however, the particle classification for the GIF:IL-2 complex (Fig. 3a) showed a drastically different assembly resembling a horseshoe-shaped structure. This prompted us to proceed to a 3D reconstruction of the particle representing the GIF:IL-2 assembly (Fig. 3c), which confirmed the shape incompatibilities with the crystal structure of the GIF:GM-CSF complex and revealed structural features to support a possible model representing the GIF:IL-2 complex (Fig. 3d). The 3D EM envelope representing the GIF:IL-2 complex can be interpreted in terms of a GIF dimer occupying the toe of the horseshoe mounted by two copies of IL-2 at the poles of the GIF dimer (Fig. 3d). The resolution of the 3D EM model did not allow us to discriminate the absolute orientation of IL-2, although the features of the 3D EM model strongly suggest that IL-2 is orientated with the longitude of its helical bundle nearly perpendicular to the plane of the GIF dimer (Fig. 3d). Indeed, similarly prepared and purified GIF:GM-CSF and GIF:IL-2 complexes characterized by SAXS yielded scattering data that despite the heterogeneous glycosylation levels of the samples, support this interpretation and further show that the two GIF–cytokine complexes have similar molecular weights (Supplementary Fig. 3a–d; Supplementary Table 3).

**Figure 4:**
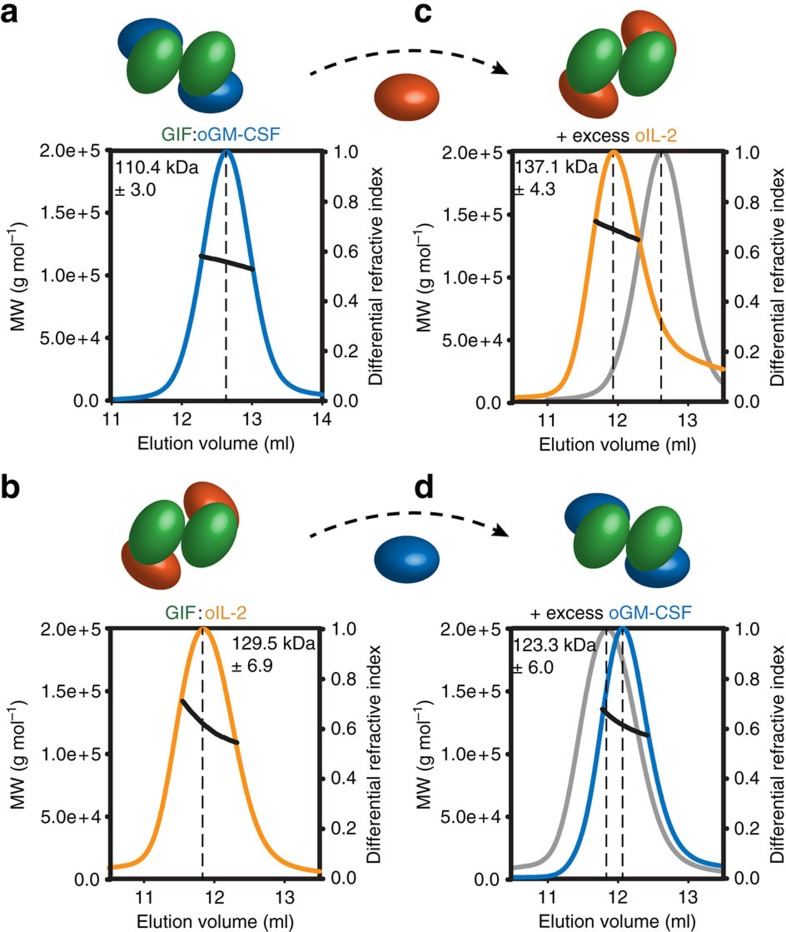
SEC-MALLS analysis of GIF:GM-CSF and GIF:IL-2 complexes. MALLS analysis of GIF:GM-CSF (**a**) and GIF:IL-2 (**b**) complexes purified after co-expression of GIF and GM-CSF/IL-2 in HEK293T cells. GIF:GM-CSF (**c**) and GIF:IL-2 (**d**) complexes re-purified after addition of a 10-fold excess of purified IL-2 or GM-CSF respectively. For each, a graph is shown plotting the differential refractive index and the derived molecular weight (grey) as a function of the elution volume after SEC. The resulting mean MW and corresponding standard deviation are presented at the top of each chromatogram.

To provide additional evidence for the molecular basis and stoichiometry of the two structurally distinct GIF–cytokine complexes we carried out a comparative analysis by SEC and MALLS (Fig. 4a,b; Supplementary Fig. 4; Supplementary Table 4). Our analyses show that both the GIF:GM-CSF and the GIF:IL-2 complexes are chromatographically relatively monodisperse as evidenced by the molecular weight profiles across each elution peak, and that both complexes obey a 2:2 stoichiometry assuming fully N-glycosylated proteins (Fig. 4a,b; Supplementary Table 4). Addition of a large molar excess of purified cytokine to its respective complex with GIF did not change the chromatographic behaviour of the complex indicating that the purified GIF–cytokine complexes were stable and fully occupied by cytokines. Along these lines and to cross-validate the consistency of the deduced 2:2 stoichiometry of the GIF:cytokine complexes, we treated a given GIF–cytokine complex with a molar excess of the second cytokine partner and analysed the resultant protein mixtures by SEC-MALLS (Fig. 4c,d). Indeed, the SEC-MALLS profiles showed that the 2:2 assemblies were conserved and that the chromatographic profiles and fits were consistent with the exchange of one cytokine for the other (Fig. 4c,d). Therefore, we conclude that GIF is able to adopt well defined 2:2 stoichiometric assemblies in the presence of GM-CSF and IL-2, despite its aggregative propensity. In this regard, we note that we were only able to observe chromatographically well-behaved GIF in the presence of detergent (0.15% (w/v) CHAPS), albeit as an unresolved mixture of dimers and tetramers (Supplementary Fig. 4a)^20^. Finally, we note that glycan analyses of the two complexes unmasked very complex N-glycan profiles suggestive of high levels of sialylation, and by extrapolation expected structural heterogeneity with respect to the glycan structures.

Thus, our structural analyses of GIF:GM-CSF and GIF:IL-2 complexes by several orthogonal approaches including X-ray crystallography, EM, SAXS and SEC-MALLS illustrate that the two GIF–cytokine complexes are structurally distinct based on the ability of dimeric GIF to sequester two copies of each cytokine to yield stable assemblies obeying 2:2 stoichiometry.

### GIF binds GM-CSF and IL-2 via mutually exclusive epitopes

**Figure 5:**
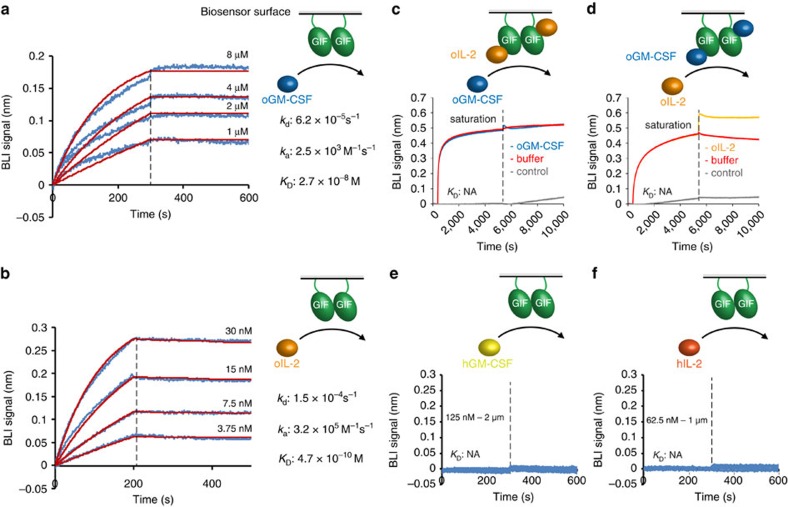
GIF binds GM-CSF and IL-2 with high affinity and distinct binding kinetic profiles. (**a**,**b**) Kinetic profiles of GIF:GM-CSF and GIF:IL-2 interactions characterized by BLI. In each case, biotinylated GIF was coupled to the surface of streptavidin coated BLI sensors, followed by binding measurements in different concentrations of GM-CSF/IL-2. Binding data was fitted using a 1:1 interaction model. (**c**,**d**) BLI measurements of the binding affinity of GM-CSF and IL-2 after saturating GIF bound sensors with IL-2 or GM-CSF respectively. After saturation with GM-CSF/IL-2, binding to the other cytokine is completely abolished (*K*_D_: NA=not applicable). (**e**,**f**) BLI measurements demonstrate that GIF does not bind to human orthologues of GM-CSF and IL-2. Displayed *K*_D_, *k*_d_ and *k*_a_ values represent the average of three replicate experiments. Errors on the average *K*_D_, *k*_d_ and *k*_a_ values can be found in Supplementary Table 5.

Previous studies demonstrated that GIF inhibits the biological activities of both ovine GM-CSF and ovine IL-2 in soft bone marrow cell colony and T-cell proliferation assays, respectively^20^. To characterize the interaction between GIF and its two target cytokines kinetically and in terms of binding affinity we employed bio-layer interferometry (BLI) using biotinylated GIF immobilized onto streptavidin-coated BLI sensors. Our measurements reveal that GIF binds GM-CSF and IL-2 with high affinities yet with quite distinct kinetic binding profiles (Fig. 5a,b). On one hand, binding of GM-CSF to GIF is characterized by rather slow association kinetics and very slow dissociation kinetics leading to a *K*_D_=27 nM, whereas IL-2 binds to GIF with ∼60-fold higher affinity (*K*_D_=0.47 nM) that can be recapitulated by fast association kinetics and moderately slow dissociation kinetics (Fig. 5a,b).

The unique capacity of GIF to bind tightly to two different cytokines resulting in structurally distinct complexes prompted us to explore the possible overlap between the GM-CSF and IL-2 binding epitopes. We thus carried out BLI experiments meant to measure the possible binding of GM-CSF and IL-2 to preassembled GIF:IL-2 and GIF:GM-CSF complexes, respectively, at concentrations that would warrant the stability of the preassembled complexes (Fig. 5c,d). We found that neither GM-CSF nor IL-2 were able to bind additively to GIF already occupied by the other cytokine, revealing that the cytokine binding sites on GIF are mutually exclusive. In light of our structural analyses, it would appear that the binding footprints of GM-CSF and IL-2 on GIF might be overlapping to a certain degree (Figs 1 and 3).

**Figure 6:**
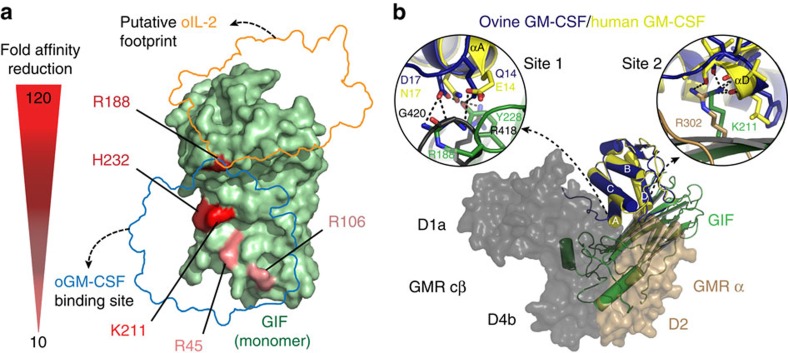
Mapping of GM-CSF and IL-2 binding footprints. (**a**) The GM-CSF and IL-2 interaction epitopes on GIF are mutually exclusive. Schematic overview of the effect of GM-CSF interface residue mutations on GIF. GIF is shown in dark green, and the GM-CSF and putative IL-2 binding sites are shown as outlines in blue and orange respectively. Mutated GIF residues (R45A, R106A, R188A, K211A and H232A) are coloured in different tints of red, depending on the resulting reduction in affinity for GM-CSF (120-fold affinity reduction: bright red, 10 fold affinity reduction: light pink). See also Supplementary Fig. 5 and Supplementary Table 5. (**b**) GIF serves as a competitive viral decoy receptor against the GM-CSF receptor. Alignment of the GIF:GM-CSF crystal structure (GIF: green, ovine GM-CSF: blue) with human GM-CSF (yellow) in the GM-CSF:GM-CSFR complex^32^ (PDB code: 3CXE, GMR α: brown, GMR cβ; grey). GM-CSF binding on GIF through α-helices αA and αD fully overlaps with GM-CSF binding epitopes on GM-CSFR α and cβ. Insets show a detailed zoom of binding interfaces to human and ovine GM-CSF helix αA (inset 1) and αD (inset 2). Residues on GIF or GM-CSFR involved in ovine/human GM-CSF binding are labeled and shown as sticks, polar interactions are shown as dashed lines.

To delineate the possible boundaries of the two distinct binding epitopes utilized by GIF to sequester GM-CSF and IL-2, we resorted to our high resolution snapshots of the GIF:GM-CSF complex to design GIF variants carrying alanine mutations at amino acids involved in the GIF:GM-CSF binding interface (Figs 1c–e and 6a). Thus, given the overwhelmingly positive electrostatic potential of the GIF-binding epitope (Fig. 1g), we hypothesized that mutation of arginine, lysine and histidine residues involved in more than one interaction with amino acids on GM-CSF along the helix-A/D face of the helical bundle might be appropriate candidate amino acids to mutate in GIF. We therefore selected to mutate Arg45, Arg106, Arg188, Lys211 and His232, and were able to produce several single- and double-mutation variants of GIF (Arg45Ala, Arg45Ala/Arg106Ala, Arg188Ala, Arg106Ala/Arg188Ala, Lys211Ala, His232Ala) as biotinylated recombinant proteins in HEK293T for binding studies via BLI (Fig. 6; Supplementary Fig. 5a–l; Supplementary Table 5).

Our BLI data revealed that all mutations caused a ∼10 to ∼120-fold decrease in the affinity of GIF for GM-CSF, with Lys211 on GIF constituting a true hotspot at the epicenter of the binding interface (Fig. 6a; Supplementary Fig. 5e; Supplementary Table 5). Interestingly, all mutations negatively affected the off-rate of the interaction, which is quite remarkable given that all mutations were alanine substitutions in the context of an interaction footprint described by very pronounced electrostatics (Fig. 1g). Such kinetic profiles lend support to the specificity of the interactions concerned. Importantly, while all but one mutation had no marked effect on the GIF:IL-2 interaction, Arg188Ala at the far end of the GIF:GM-CSF interaction epitope bordering the putative IL-2 binding site (Fig. 1c) elicited a 10-fold reduction in the affinity of the GIF:IL-2 interaction and a near 20-fold decrease in the affinity of the GIF:GM-CSF interaction by drastically impacting the interaction off-rate (Fig. 6a; Supplementary Fig. 5i,f; Supplementary Table 5). In light of our entire alanine-scanning mutagenesis data set, this very fortuitous observation lends strong support to the localization of the IL-2-binding footprint as modeled immediately adjacent to, and quite possibly partly overlapping with, the GM-CSF binding site (Fig. 6a). Indeed, such mode of binding is corroborated well by the mutual exclusivity of the two cytokine binding sites revealed by our binding studies using wild-type GIF (Fig. 5c,d), because the presence of one cytokine on GIF would sterically prevent binding of the other.

### GIF interferes competitively with cytokine–receptor interfaces

Given the observed high potency by which GIF specifically neutralizes ovine GM-CSF and IL-2 (Fig. 5c,d) but not the human counterparts (Fig. 5e,f) and in light of the GIF-mediated abrogation of the bioactivity of the two cytokines in cellular assays^20^, we sought to trace the structural basis for the antagonistic properties of GIF. In the first instance we compared the binding mode of ovine GM-CSF to GIF with the structure of human GM-CSF in complex with its cognate receptors GMRα and βc (refs 32, 33). The two GM-CSF orthologues share 80% sequence identity and can be superposed with an r.m.s.d. of 0.7 Å (backbone atoms). Our analyses show that binding of GIF to GM-CSF drastically overlaps with both cognate binding epitopes of GM-CSF to GMRα and β_c_ (Fig. 6b). Consequently, GIF can be classified at the outset as a competitive decoy receptor for GM-CSF.

A closer look at the structural superimpositions reveals that competitive antagonism by GIF goes well beyond a steric exclusion mechanism. Remarkably, GIF engages several residues in helices αA and αD of GM-CSF that would be involved in binding to GMRα (Fig. 6b, sites 1 and 2). In particular we highlight the deployment of Lys 211 by GIF to cap helix αD in GM-CSF (Fig. 6b, site 2), which is exactly what the functionally important Arg302 of GMRα does^34^,^35^ in the GMRα:GM-CSF complex^32^. Furthermore, GIF employs a triad of residues (Gln 108, Tyr 110 and Arg 45) to engage residue Asp112 in GM-CSF, which constitutes a structural and functional hotspot for interacting with GMRα (refs 34, 35, 36). Together, these analyses show that GIF has adopted a mechanism of interface mimicry^37^ and illustrate how a viral protein bearing no sequence or structural similarity to the cognate receptors of the target cytokine has evolved convergently to sequester the target cytokine by partially hijacking key principles and interactions underlying the cognate cytokine–receptor complex. Indeed, such intricate mimicry might present a daunting challenge to the host in terms of developing an effective counter.

Regrettably, the lack of structural information at high resolution for the binding of IL-2 to GIF limits us from making a robust proposal for the possible structural mechanism underpinning the role of GIF as a viral decoy receptor to antagonize IL-2-mediated signalling in the host. However, we note that the likely near vertical orientation of the long axis of the helical bundle of IL-2 with respect to the GIF dimer (Fig. 3d) in the context of IL-2 in complex with IL-2Rα, IL-2Rβ and γ_c_ (ref. 38), would create severe steric hindrance for binding of either the IL-2Rβ or the γ_c_ receptor chains. Interestingly, it is well established that binding of IL-2 to IL-2Rα primes IL-2 for binding with a higher affinity to IL-2Rβ (refs 38, 39). It is thus tempting to consider whether GIF might play into the mechanism of IL-2-mediated receptor activation by binding the activated form of IL-2 with high affinity to compete more effectively against the cognate cytokine receptors.

## Discussion

**Figure 7:**
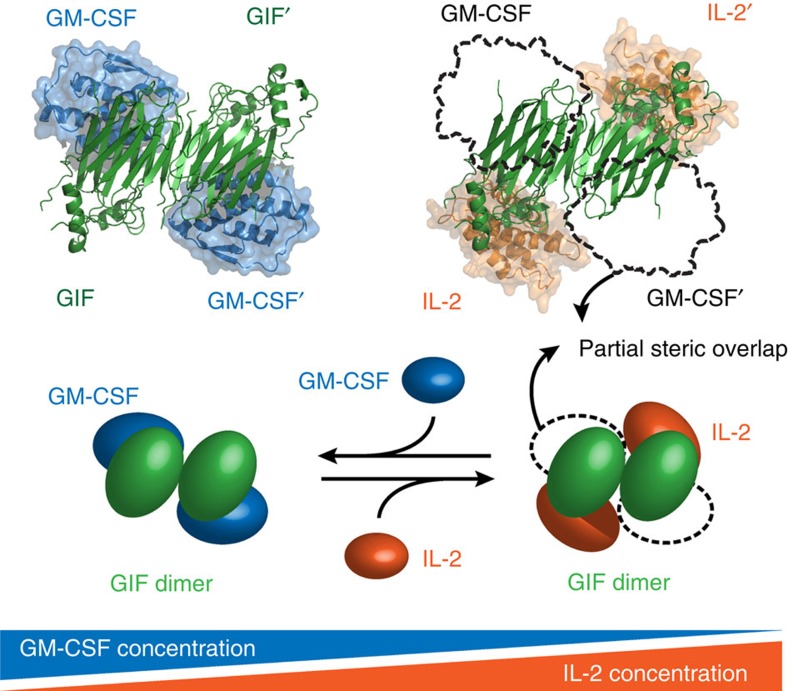
Schematic mechanistic recapitulation of the two possible GIF–cytokine complexes. GM-CSF (dark blue) and IL-2 (orange) have partially overlapping binding sites on GIF (dark green). In the presence of an excess of IL-2, the GIF:GM-CSF complex switches to a GIF:IL-2 complex, and vice versa.

The Orf virus GIF protein provides a fascinating example of viral protein evolution to enable evasion of the host immune response. A decade and a half after the discovery of GIF as a viral protein able to antagonize the bioactivity of both GM-CSF and IL-2 (ref. 20), two distinct cytokines pivotal to the host immune response, we have presented here the structural and mechanistic determinants underlying cytokine sequestration by GIF. GIF neutralizes GM-CSF and IL-2 with high affinity by engaging the two cytokines on mutually exclusive binding epitopes on the concave surface of its dimeric scaffold yielding two structurally distinct complexes (Fig. 7). The structural and evolutionary origins of GIF can be traced to a conserved beta-sandwich fold that serves as the hallmark of the PIE domain superfamily^40^. Although GIF does not bear any resemblance to the cognate receptors of GM-CSF and IL-2 whatsoever, it targets hotspots and mechanistic binding principles that are pivotal to the assembly of productive complexes between GM-CSF and IL-2 and their cognate receptors. In this regard, GIF joins a very sparsely populated class of viral decoy cytokine receptors that are structurally distinct from the cognate receptors, as was also recently shown for BARF1 from the Epstein-Barr virus^41^, and the Yaba-like disease virus 2L protein^42^.

**Figure 8:**
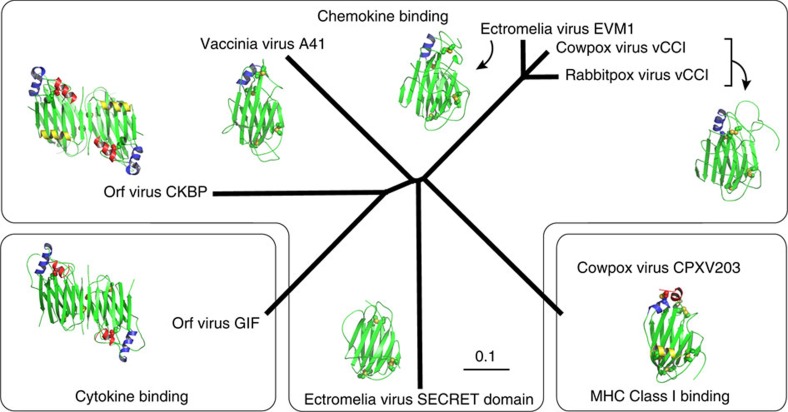
Phylogenetic tree of members of the Poxviral Immune Evasion (PIE) superfamily with known structures. The phylogenetic tree, based on the alignment presented in Supplementary Fig. 1, is generated by ClustalW2 (ref. 78) and visualized by Geneious 4.8.5 (ref. 79). The scale bar represents the number of substitutions per site. Representative structures (Orf virus GIF: 5D28, Orf virus CKBP: 4ZK9 , Vaccinia virus A41: 2VGA, Rabbitpox virus vCCI: 2FFK, ectromelia virus SECRET domain: 3ON9 , Rabbitpox virus CPXV203: 4HKJ) displaying a variation of the PIE superfamily β-sandwich fold are shown for each branch.

The herein structural characterization of GIF now provides the structural prototype for PIE viral proteins that target cytokines, defining a distinct functional branch in the phylogenetic tree of the PIE superfamily (Fig. 8). Most structurally characterized members of the PIE superfamily (vaccinia virus A41L (ref. 27), ectromelia virus EVM1 (ref. 30), cowpox virus vCCI (ref. 28), rabbitpox vCCI (ref. 29), Orf virus CKBP (ref. 31), and the SECRET domain of the ectromelia virus CrmD^43^,^44^) are functionally annotated as *bona fide* chemokine binding proteins, while a third functional branch is represented by a soluble MHC Class 1-binding protein from the cowpox virus CPXV203 (ref. 45). A similar β-sandwich fold, albeit with a different β-strand topology, is also found in the N-terminal domain of M3, a chemokine binding protein secreted by murine γ-herpesvirus 68 (ref. 46).

**Figure 9:**
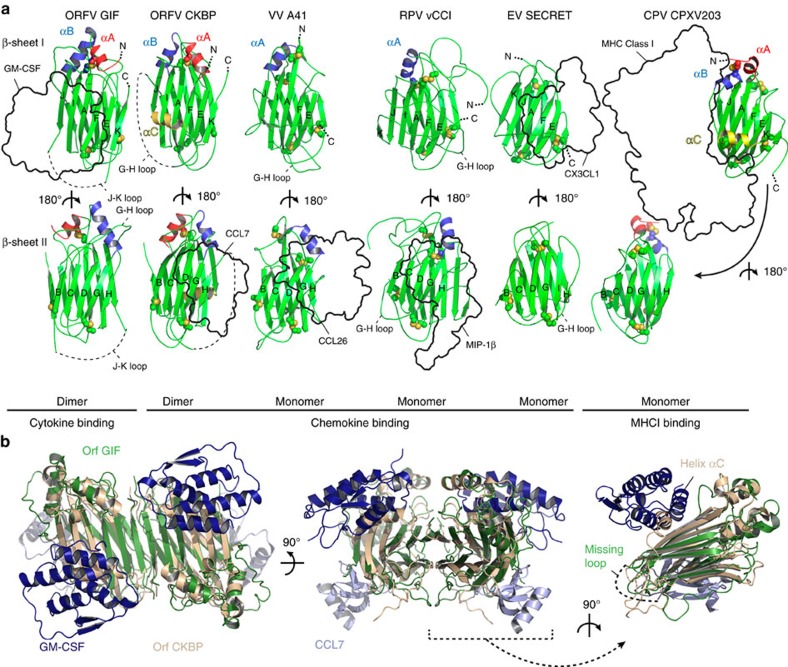
Structural comparison of Orf GIF with other members of the Poxviral Immune Evasion (PIE) superfamily. (**a**) Structures for Orf virus GIF (PDB code 5D28),Orf virus CKBP (PDB code: 4ZK9), Vaccinia virus A41 (PDB code: 2VGA), Rabbitpox virus vCCI (PDB code: 2FFK), Ectromelia virus SECRET domain (PDB code: 3ON9) and Cowpox virus CPXV203 (PDB code: 4HJK) are shown in cartoon representation with labeled secondary structure elements and disulfides shown as spheres. Binding ligands are labeled and shown as a black outline. Shared secondary structure elements consist of 2 β-sheets (I: β-strands βJ, βI, βA, βF, βE, βK and II: β-strands βB, βC, βD, βG, βH) and one common α-helix linking β-strands βF and βG present in all proteins except the SECRET domain. Within the β-sandwich, six cysteines are fully conserved throughout all PIE superfamily members, resulting in three disulfide bonds stabilizing the β-sandwich core. The related Orf GIF and Orf CKBP proteins contain one and two extra α-helices, respectively, which are not present in the folds of vaccinia virus A41, rabbit pox virus vCCI and ectromelia virus SECRET domain. However, cowpox virus CPXV203 contains two extra C-terminal α-helices of which the second one is present at an equivalent position as the C-terminal helix observed in Orf CKBP. (**b**) Structural alignment of GIF from the GIF:GM-CSF complex (GIF: green, GM-CSF: dark blue) with Orf CKBP bound to CCL7 (CKBP: wheat, CCL7: light blue).

With such a wealth of structural data at hand we deemed it opportune to trace the common and differentiating structural features of the PIE superfamily (Fig. 9). The stunning versatility of the common β-sandwich core becomes apparent when comparing the targeting (CC and CXC chemokines, cytokines, MHC class I molecules) as well as the binding epitope of ligands binding the aforementioned proteins. Whereas Orf CKBP, A41 and vCCI all bind chemokines on the acidic surface formed by β-sheet II, Orf GIF binds both GM-CSF and IL-2 through a basic patch present on β-sheet I (Fig. 9a). Moreover, although the surface charge distribution of β-sheets I and II is generally conserved among PIE superfamily members, the ectromelia virus SECRET domain displays reversed surface charge properties of β-sheets I and II, and differs from other viral CKBPs since it binds chemokines on the negatively charged surface of β-sheet I. Conversely, β-sheet I is shielded away by two loops in A41 and vCCI: one connecting β-strands βG and βH, and the second present at the C-terminal end of both proteins. The βG–βH loop serves a different role in Orf GIF, where it is involved in GM-CSF binding through the functionally important Arg188 (Fig. 1c) as well as stabilizing the GIF dimer through binding to the βG′–βH′ loop of an opposing GIF monomer. In Orf CKBP, β-sheet I is occupied by the extra α-helix αC, which connects β-strands βJ and βK. Cowpox virus CPXV203 on the other hand uses the extra C-terminal helices αB and αC covering β-sheet I to bind into a groove formed by subunits α2 and α3 of MHC1 (Fig. 9a). Helix αC is not present in Orf GIF, instead it is replaced by an arginine- and proline-rich stretch of 15 amino acids connecting βJ and βK. This loop region is predicted to be unstructured and in fact we were not able to model it in the electron density maps for the GIF:GM-CSF complex. Phylogenetic analysis suggests that the binding specificity of GIF towards ovine GM-CSF and IL-2 most likely appeared after speciation of the Orf virus^16^. It thus appears that the J-K loop diverged to an α-helix in Orf CKBP and an unstructured loop in Orf GIF, which allows the surface formed by β-sheet I to bind GM-CSF and IL-2 (Fig. 9b).

An intriguing aspect of the PIE superfamily β-sandwich fold concerns its oligomerization capacity. A41, vCCI, CPXV203 and the ectromelia virus SECRET domain form monomers in solution, while Orf CKBP is an obligate dimer (Fig. 9a). Interestingly, the SECRET domain present in the CrmB gene from variola virus was also found to be a dimer^47^. Orf CKBP dimerizes via β-sheet complementation involving its C-terminal β-strand βK. A similar interface involving β-strand βK is found in GIF dimers, where the binding between GIF monomers is further strengthened by additional interactions mediated by the G-H loop. By combining an oligomeric state with binding multivalence on a single molecular platform that can engage the target molecules with high affinity into long-lived complexes, GIF appears to recapitulate well the general strategy employed by viral effector proteins and decoy receptors for inflicting a potent and efficient modulation of the host immune system within the relatively short timeframes available to them^41^,^48^. Arguably, what appears to set GIF apart from all other viral decoy receptors characterized to date is not only its ability to target two different cytokines but also the fact that it does so via structurally distinct assemblies (Fig. 7). In the case of GM-CSF, the mechanistic consequences of its sequestration by GIF can be traced to interference with the cognate cytokine receptor binding interfaces (Fig. 6b), which is likely the mechanism deployed for the antagonism of IL-2 activity as well. Together, our work contributes a novel example of the virtuosity and potency of viral effector proteins and molecular decoys to achieve immunomodulation of the host.

## Methods

### Protein production and purification

Sequence optimized constructs containing residues 18–144 of ovine GM-CSF (Uniprot code: P28773) and residues 21–155 of ovine IL-2 (Uniprot code: P19114) were cloned in the pET-15b vector (Novagen) and pHLsec vector^49^ encoding an N and C-terminal His_6_-tag respectively. Ovine GM-CSF and IL-2 cloned in the pET-15b vector were expressed in BL21(DE3) (Novagen) *Escherichia coli* cells and produced from inclusion bodies by rapid dilution refolding (described in reference Verstraete *et al*.^50^), while the same constructs cloned in the pHLsec vector were transiently expressed in mammalian HEK293T cells^49^. Refolded oGM-CSF and oIL-2 were purified by IMAC using prepacked 5 ml Ni-NTA Superflow cartridges (QIAGEN). After binding, oGM-CSF or oIL-2 were washed with 10 column volumes washing buffer (50 mM NaH_2_PO_4_, 300 mM NaCl, 15 mM Imidazole pH 7.0) and eluted with 10 column volumes elution buffer (50 mM NaH_2_PO_4_, 300 mM NaCl, 500 mM Imidazole, pH 7.0). After elution protein fractions were pooled, concentrated to a volume of 1 ml and injected onto a Prep-Grade Hiload 16/600 SD 75 size exclusion column (GE Healthcare) equilibrated with HEPES-buffered saline buffer (HBS, 20 mM HEPES, pH 7.5, 150 mM NaCl). oGM-CSF and oIL-2 expressed in HEK293T cells were purified using the same purification protocol as described above, but instead of Ni-NTA cartridges, a Co^2+^-packed TALON FF column (Clontech) was used for IMAC.

A construct containing residues 1-265 of the Orf virus GM-CSF/IL-2 inhibition factor (GIF, Uniprot code: Q9J5U5) was cloned into the pHLsec vector encoding a C-terminal His_6_-tag^49^. His-tagged GIF was transiently expressed in HEK293T cells. During transfection, sodium valproic acid (3.6 mM) was added to the transfection medium to boost expression levels. After 5 days of expression, medium was harvested, supplemented with 0.15% (weight per volume) CHAPS and loaded onto a Co^2+^-packed TALON FF column (Clontech). During IMAC, the same buffers as described above were used, supplemented with 0.15% (weight per volume) CHAPS. Eluted GIF was pooled, concentrated and injected onto a Prep-Grade Hiload 16/600 SD 200 size exclusion column (GE healthcare) equilibrated with HBS supplemented with 0.15% (weight per volume) CHAPS.

To generate GIF:oGM-CSF and GIF:oIL-2 complexes, GIF was either co-expressed with oGM-CSF or oIL-2 in HEK293T cells by transient transfection^49^, or purified after addition of an excess or refolded oGM-CSF or oIL-2 to the HEK293T expression medium containing GIF. In each case, expression medium was harvested after 5 days of co-expression and loaded onto a Co^2+^-packed TALON FF column (Clontech) or Ni^2+^-packed complete column (Roche) for oIL-2 and oGM-CSF, respectively. Subsequent IMAC and SEC purification steps were performed using the protocol as described for unliganded GIF, but without adding CHAPS to purification buffers.

### Crystallization and data collection

All crystallization trials were set up using a Mosquito robot (TTP LabTech) in a sitting drop vapour diffusion set-up with drop volumes of 200 nl (100 nl protein solution+100 nl reservoir solution). Refolded oGM-CSF was concentrated to 5 mg ml^−1^ before initiating crystallization trials. Large plate-like crystals were obtained at 293 K in a crystallization condition containing 0.2 M sodium acetate trihydrate, 0.1 M TRIS hydrochloride pH 8.5 and 30% (weight per volume) PEG 4,000 (Hampton Crystal Screen 1, condition B10). Crystals were scooped, transferred to mother liquor supplemented with 25% ethylene glycol, and subsequently flash-cooled in liquid nitrogen. Diffraction data were collected to 2.0 Å at the P14 microfocus beam line at PETRA III, Hamburg. The obtained data set was processed using XDS^51^.

Purified complex between GIF produced in HEK293T cells and *in vitro* refolded oGM-CSF produced in *E. coli* was concentrated to 6 mg ml^−1^. Small and highly mosaic cube-like crystals were obtained at 293 K in a crystallization condition containing 0.15 M ammonium sulfate, 0.1 M MES pH 5.5 and 25% (weight per volume) PEG4000 (Molecular Dimensions ProPlex screen, condition C10). After optimization of this initial hit, new crystals with dimensions of 40 × 40 × 250 μm slowly grew over a period of 3 months in a condition containing 0.15 M ammonium sulfate, 0.1 M MES pH 6 and 24% (weight per volume) PEG 4,000. Before X-ray data collection, crystals were scooped, transferred to mother liquor supplemented with 25% ethylene glycol, and flash cooled in liquid nitrogen. Diffraction data were collected to 2.84 Å at the P14 microfocus beam line at PETRA III, Hamburg, and subsequently processed using XDS^51^. An overview of all data collection and refinement statistics can be found in Table 1.

### Structure determination and refinement

Diffraction data for ovine GM-CSF was processed in spacegroup *P*2_1_ (*a*=41.1 Å, *b*=77.02 Å, *c*=47.45 Å, *β*=111.5°). Structure determination was performed by maximum-likelihood molecular replacement (MR) in Phaser^52^ using the crystal structure of human GM-CSF (PDB code 2GMF^53^) as a search model. The resulting single MR solution was refined in Phenix^54^ using positional and real-space refinement with non-crystallographic symmetry (NCS) restraints, individual anisotropic atomic displacement parameter (ADP) refinement and optimized X-ray/stereochemistry and X-ray/ADP weights (Table 1; Supplementary Fig. 6).

The obtained data set for the GIF:oGM-CSF complex was processed in space group *C*2 (*a*=145.8 Å, *b*=105.7 Å, *c*=73.5 Å, *β*=93.3°). After manual inspection of the collected data, severe anisotropy was observed. The data were ellipsoidally truncated and anisotropically scaled on-line using the anisotropy server^55^, which recommended resolution limits along the a*, b* and c* axes of 3.3 Å, 2.8 Å and 2.7 Å, respectively. As no homologues of the Orf GIF protein are available, a trimmed ensemble model was made with the program Ensembler of the Phenix package by using two proteins distantly related to GIF, vaccinia virus A41L (ref. 27) (PDB code=2VGA, 19% sequence identity) and cowpox virus vCCI (ref. 28) (PDB code=1CQ3, 11% sequence identity). This trimmed ensemble and the refined structure of ovine GM-CSF, were used as search models for maximum-likelihood MR in Phaser^52^. This led to an MR solution containing two trimmed ensembles and two oGM-CSF molecules, whereby a GIF dimer is formed decorated by two oGM-CSF molecules at equivalent positions on GIF. Initial refinement cycles were performed in Buster^56^ using NCS refinement and anisotropic ADP refinement with TLS. After tracing of the main chain and most of the missing side-chains of GIF, further refinement cycles were carried out in Phenix using positional and real-space refinement with NCS restraints, individual anisotropic ADP refinement combined with TLS and optimized X-ray/stereochemistry weights (Table 1; Supplementary Fig. 6).

### Production of biotinylated proteins for kinetic studies

Residues 1–265 of Orf GIF as well as six interface residue mutant GIF species (R45A, R45A/R106A, R188A, R106A/R188A, K211A and H232A) were cloned in the pHLsec vector containing a C-terminal AviTag^49^. The pHLsec-GIF-AviTag constructs were transiently co-transfected in HEK293T cells with *E. coli* biotin ligase (BirA) cloned in the pDisplay vector (pDisplayBirA-ER^57^). Before co-transfection, cell medium was replaced by serum-free DMEM medium containing 100 μM of D-biotin. After 5 days of co-expression, expression medium was harvested and desalted using a HiPrep 26/10 Desalting column (GE Healthcare) equilibrated with HBS to remove excess D-biotin.

### Bio-layer interferometry

All experiments were performed in 1 × PBS (137 mM NaCl, 2.7 mM KCl, 10 mM Na_2_HPO_4_, 1.8 mM KH_2_PO_4_, pH 7.4) supplemented with 0.01% (w/v) bovine serum albumin and 0.002% Tween-20, using an Octet RED96 instrument (ForteBio), operating at 298 K. Streptavidin-coated biosensors were functionalized with biotinylated GIF, quenched with a 10 μg ml^−1^ biocytin solution and then exposed to different concentrations of ligand (GM-CSF: 1–8 μM, IL-2: 3.75–30 nM). To verify that no nonspecific binding was present during the interaction assay, non-functionalized biosensors were used as a control by measuring in parallel all ligand concentrations as well as running buffer. The measurements presented in Fig. 5c were carried out based on sensor tips bound with immobilized GIF saturated with 250 nM of oIL-2 and were used for measurements against 20 μM oGMCSF. The measurements presented in Fig. 5d were carried out based on sensor tips bound with immobilized GIF saturated with 20 μM oGMCSF and were used for measurements against 250 nM of oIL-2. All data were fitted with the FortéBio Data Analysis 7.1 software using a 1:1 interaction model. All binding experiments were performed in triplicate. Displayed *K*_D_, *k*_d_ and *k*_a_ values represent the averages of these triplicates.

### Negative-stain electron microscopy and image analysis

GIF:IL-2 was applied to the clean side of carbon on a carbon–mica interface and stained with 2% (weight per volume) uranyl acetate. Images were recorded under low-dose conditions with a JEOL 1,200 EX II microscope at 100 kV and at a nominal magnification of 40,000x. Negatives were digitized on a Zeiss scanner (Photoscan TD) to a pixel size of 3.5 Å at the object scale (Supplementary Fig. 2a). A semi-automatic particle selection with BOXER^58^ led to an extraction of a total of 26,910 GIF:IL-2 subframes of 64 × 64 pixels. CTF correction and 2D reference-free classification was performed in RELION^59^ (Supplementary Fig. 2b). 2D class averages suggested the presence of a two-fold symmetry axis. Three different sets of 30 to 40 best class averages were extracted and used to calculate 12 *ab initio* 3D reconstructions of GIF:IL-2 with VIPER applying a C2 symmetry^60^,^61^,^62^. All plausible solutions had a similar horseshoe appearance with a well-defined global core. Therefore, they were aligned in 3D, averaged and filtered to 50 Å to create a low resolution GIF:IL-2 3D model (Supplementary Fig. 2c). This model was further refined in RELION using 3D auto-refinement and 3D classification procedures (Supplementary Fig. 2d). In an attempt to better take the SAXS data into account, a SAXS model of GIF:IL2 was filtered to 50 Å resolution and used as an initial model for another cycle of RELION refinement of the previously selected 24,324 GIF:IL-2 particles. This final refinement resulted in 3D maps with a resolution of 17 Å according to the FSC=0.5 criterion (Supplementary Fig. 2e,f). The final map was filtered to 25 Å resolution.

GIF:GM-CSF was applied to the clear side of carbon on a carbon–mica interface and stained with 2% (w/v) sodium silicotungstate, pH 7.5. Images were recorded under low-dose conditions with a FEI Tecnai12 microscope operating at 120 kV and at a nominal magnification of 49,000 × using a Gatan Orius SC1,000 CCD camera. The CTF for each micrograph was determined with CTFFIND3 (ref. 63). Using projections of the crystal structure of GIF-GM-CSF filtered to 18 Å as templates, particles were automatically selected from all the micrographs using the Fast Projection Matching (FPM) algorithm^64^. The resulting data set was cleaned for bad images. The final 73,153 particles were filtered to 25 Å and two-dimensional (2D) reference-free classification was performed in RELION^63^ with no CTF correction.

### Modeling of GIF and oIL-2 in the EM map

The crystal structure of dimeric GIF and two copies of an oIL-2 homology model generated by MODELLER^65^ were fitted in the EM map of the GIF:oIL-2 complex using the autofit procedure in UCSF Chimera^66^.

### Small-angle X-ray scattering

SEC-SAXS data were collected at the SWING beamline at SOLEIL (France) using an integrated online HPLC set-up. Purified samples of GIF:oGM-CSF and GIF:oIL-2 co-expressed in HEK293T cells were first concentrated to 10 and 8.8 mg ml^−1^ respectively before injection on a Superdex Increase 10/300 GL size exclusion column (GE Healthcare) equilibrated with HBS (pH 7.5) using an injection volume of 100 μl and a flow rate of 0.5 ml min^−1^. Measurements were performed at 293 K, within a momentum transfer range of 0.01 Å^−1^<s<0.550 Å^−1^ (GIF:GM-CSF) and 0.01 Å^−1^<s<0.622 Å^−1^ (GIF:IL-2), where *s*=4*π*sin(*θ*)/*λ* and 2*θ* is the scattering angle. Eighty nine buffer frames with an exposure time of 1 s were averaged and subtracted from 10 × 1 s frames (GIF:GM-CSF) or 16 × 1 s frames (GIF:IL-2) taken from the eluted protein peaks. Scattering data were collected on an AVIEX PCCD170170 detector. Initial data integration, averaging and subtraction were performed using the Foxtrot software at SWING. The forward scattering (*I*_0_) and radius of gyration (*R*_g_) were determined by PRIMUS-QT^67^ using Guinier approximation^68^, and compared with values for *I*_0_ and *R*_g_ calculated by SCÅTTER^69^. The Porod volume estimate (*V*_p_) was evaluated using Autoporod^70^, the maximum particle dimension *D*_max_ and corresponding distance distribution function *P*(r) were evaluated using SCÅTTER^69^. Molecular masses of GIF:oGM-CSF and GIF:oIL-2 complexes were calculated using methods independent of calibration standards provided by the SAXSMoW^71^ and SCÅTTER^69^ software. An overview of SAXS data collection, structural parameters and MW determination can be found in Supplementary Table 3.

### Modeling of SAXS data

The crystal structure of the GIF:oGM-CSF complex and the model of GIF:IL-2 based on the fit in the EM map were used as an input for the Allosmod-FoXS web server^72^ to model missing loops, N and C termini and N-linked glycosylation. During each Allosmod-FoXS run, data up to a scattering angle of 0.5 Å^−1^ was used. Fits of the resulting SAXS models of GIF:GM-CSF and GIF:IL-2 complexes to the experimental SAXS data were calculated using the FoXS software^73^.

### Multi-angle laser light scattering

Purified oGM-CSF, oIL-2, GIF:oGM-CSF and GIF:oIL-2 samples expressed in HEK293T cells were concentrated to 1–2 mg ml^−1^ and injected onto a Superdex Increase 10/300 GL size exclusion column (GE Healthcare) equilibrated with HBS (pH 7.5), coupled to an online UV-detector (Shimadzu), a mini DAWN TREOS (Wyatt) multi-angle laser light scattering detector and an Optilab T-rEX refractometer (Wyatt) at 298 K. Following refractive index (RI) increment values (d*n*/d*c*) were used for protein concentration and molecular mass determination: ovine GM-CSF: 0.182 ml g^−1^, ovine IL-2: 0.182 ml g^−1^, GIF:oGM-CSF: 0.181 ml g^−1^, GIF:oIL-2: 0.182 ml g^−1^. Data analysis was carried out using the ASTRA6.1 software. Theoretical and measured molecular weights relevant to this study are summarized in Supplementary Table 4.

### Data availability

Coordinates and structure factors for ovine GM-CSF and the GIF:GM-CSF complex have been deposited in the Protein Data Bank (PDB) with accession codes 5D22 and 5D28 respectively. EM maps obtained after 3D reconstruction of the GIF:IL-2 complex have been deposited in the Electron microscopy Data Bank (EMDB) with accession code EMD-3230. Experimental SAXS data, a SAXS model for the GIF:GM-CSF and GIF:IL-2 complexes after loop and N-glycan modeling in Allosmod-FoXS, and the fit with experimental SAXS curves have been deposited in the Small-Angle Scattering Biological Data Bank (SASBDB) with accession codes SASDA89 (GIF:GM-CSF) and SASDA99 (GIF:IL-2).

## Additional information

**How to cite this article:** Felix, J. *et al*. Structural basis of GM-CSF and IL-2 sequestration by the viral decoy receptor GIF. *Nat. Commun.* **7,** 13228 doi: 10.1038/ncomms13228 (2016).

## Supplementary Material

Supplementary Information Supplementary Figures 1-6 and Supplementary Tables 1-5.

## Figures and Tables

**Table 1:** Crystallographic data collection and refinement statistics.

	**GIF:oGM-CSF**	GIF:oGM-CSF, anisotropy corrected*	**oGM-CSF**
Data collection statistics			
Beamline	P14 (PETRA III, Hamburg)	P14 (PETRA III, Hamburg)	P14 (PETRA III, Hamburg)
Space group	*C2*	*C2*	*P2*_*1*_
Cell dimensions			
*a*, *b*, *c* (Å)	145.8, 105.7, 73.5	145.8, 105.7, 73.5	41.1, 77.0, 47.5
*α*, *β*, *γ* (°)	90.0, 93.3, 90.0	90.0, 93.3, 90.0	90.0, 111.5, 90.0
Resolution (Å)	50.0–2.8 (3.0–2.8)	50.0–2.8 (3.0–2.8)	38.51–2.0 (2.1–2.0)
Unique reflections	25798 (4019)	21262 (1126)	18252 (2825)
*R*_meas_ (%)	7.3 (112.1)	5.9 (53.5)	12.9 (73.2)
〈*I*/*σ*(*I*)〉	20.4 (1.8)	23.2 (3.6)	8.2 (2.1)
CC(1/2)	99.9 (83.2)†	99.9 (83.2)†	98.7 (38.0)†
Completeness (%)	98.2 (95.7)	85.4 (25.1)	96.9 (94.0)
Multiplicity	6.9 (6.7)	5.9 (1.6)	3.3 (3.1)
Wilson B-factor (Å^2^)	86.4	58.3	25.7
			
Refinement			
Resolution (Å)	50.0–2.84	50.0–2.84	38.51–1.99
*R*_work_/*R*_free_	0.196/0.236‡	0.192/0.235	0.188/0.229
No. atoms	5,606	5,606	2,055
Protein	5,395	5,395	1,922
Glycan	184	184	—
Solvent	27	27	133
Average B factor (Å^2^)	62.1	62.1	34.5
Protein	61.0	61.0	34.1
Glycan	95.2	95.2	—
Solvent	39.6	39.6	39.8
R.m.s. deviations			
Bonds (Å)	0.007	0.007	0.009
Angles (°)	0.925	0.925	1.068
Ramachandran favored (%)	96.3	96.3	98.3
Ramachandran outliers (%)	0	0	0
PDB access code	5D28‡	5D28	5D22

Values in parentheses correspond to the highest-resolution shell.

CC(1/2)=percentage of correlation between intensities from random half-data sets^80^.

^*^Ellipsoidal truncation and anisotropic scaling of the data were performed using the Diffraction Anisotropy Server^55^.

^†^Correlation significant at the 0.1% level.

^‡^Structure 5D28 (refined against anisotropy corrected data) validated against full data range.

## References

[b1] Tortorella D., Gewurz B. E., Furman M. H., Schust D. J. & Ploegh H. L. Viral subversion of the immune system. Annu. Rev. Immunol. 18, 861–926 (2000). 10837078 10.1146/annurev.immunol.18.1.861

[b2] Iannello A. . Viral strategies for evading antiviral cellular immune responses of the host. J. Leukoc. Biol. 79, 16–35 (2006). 16204622 10.1189/jlb.0705397

[b3] Alcami A. Viral mimicry of cytokines, chemokines and their receptors. Nat. Rev. Immunol. 3, 36–50 (2003). 12511874 10.1038/nri980

[b4] Epperson M. L., Lee C. A. & Fremont D. H. Subversion of cytokine networks by virally encoded decoy receptors. Immunol. Rev. 250, 199–215 (2012). 23046131 10.1111/imr.12009 PMC3693748

[b5] Heidarieh H., Hernaez B. & Alcami A. Immune modulation by virus-encoded chemokine binding proteins. Virus Res. 209, 67–75 (2015). 25791735 10.1016/j.virusres.2015.02.028

[b6] Haig D. M., Hutchinson G., Thomson J., Yirrell D. & Reid H. W. Cytolytic activity and associated serine protease expression by skin and afferent lymph CD8+ T cells during orf virus reinfection. J. Gen. Virol. 77, 953–961 (1996). 8609492 10.1099/0022-1317-77-5-953

[b7] Haig D. M. & Mercer A. A. Ovine diseases. Orf. Vet. Res. 29, 311–326 (1998). 9689744

[b8] Hosamani M., Scagliarini A., Bhanuprakash V., McInnes C. J. & Singh R. K. Orf: an update on current research and future perspectives. Expert Rev. Anti-Infect. Ther. 7, 879–893 (2009). 19735227 10.1586/eri.09.64

[b9] Ara M. . Giant and recurrent orf virus infection in a renal transplant recipient treated with imiquimod. J. Am. Acad. Dermatol. 58, S39–S40 (2008). 18191701 10.1016/j.jaad.2006.04.027

[b10] Scagliarini A. . Antiviral activity of HPMPC (cidofovir) against orf virus infected lambs. Antiviral Res. 73, 169–174 (2007). 17049627 10.1016/j.antiviral.2006.09.008 PMC1930164

[b11] Perry B. D., Randolph T. F., McDermott J. J., Sones K. R. & Thornton P. K. Investing in Animal Health Research to Alleviate Poverty. International Livestock Research Institute: Nairobi, Kenya, (2002).

[b12] Jenkinson D. M., Hutchison G. & Reid H. W. The B and T cell responses to Orf virus infection of ovine skin. Vet. Dermatol. 3, 57–64 (1992).

[b13] Lear A., Hutchison G., Reid H. W., Norval M. & Haig D. M. Phenotypic characterisation of the dendritic cells accumulating in ovine dermis following primary and secondary orf virus infections. Eur. J. Dermatol. 6, 135–140 (1996).

[b14] Haig D. M. & McInnes C. J. Immunity and counter-immunity during infection with the parapoxvirus orf virus. Virus Res. 88, 3–16 (2002). 12297324 10.1016/s0168-1702(02)00117-x

[b15] Haig D. M. & Fleming S. Immunomodulation by virulence proteins of the parapoxvirus orf virus. Vet. Immunol. mmunopathol. 72, 81–86 (1999). 10.1016/s0165-2427(99)00119-1 10614496

[b16] Seet B. T. . Analysis of an orf virus chemokine-binding protein: shifting ligand specificities among a family of poxvirus viroceptors. Proc. Natl Acad. Sci. USA 100, 15137–15142 (2003). 14657392 10.1073/pnas.2336648100 PMC299921

[b17] Haig D. M. . A comparison of the anti-inflammatory and immuno-stimulatory activities of orf virus and ovine interleukin-10. Virus Res. 90, 303–316 (2002). 12457984 10.1016/s0168-1702(02)00252-6

[b18] Lyttle D. J., Fraser K. M., Fleming S. B., Mercer A. A. & Robinson A. J. Homologs of vascular endothelial growth factor are encoded by the poxvirus orf virus. J. Virol. 68, 84–92 (1994). 8254780 10.1128/jvi.68.1.84-92.1994 PMC236267

[b19] Haig D. M. . The orf virus OV20.0L gene product is involved in interferon resistance and inhibits an interferon-inducible, double-stranded RNA-dependent kinase. Immunology 93, 335–340 (1998). 9640243 10.1046/j.1365-2567.1998.00438.x PMC1364081

[b20] Deane D. . Orf virus encodes a novel secreted protein inhibitor of granulocyte-macrophage colony-stimulating factor and interleukin-2. J. Virol. 74, 1313–1320 (2000). 10627542 10.1128/jvi.74.3.1313-1320.2000 PMC111466

[b21] Hamilton J. A. Colony-stimulating factors in inflammation and autoimmunity. Nat. Rev. Immunol. 8, 533–544 (2008). 18551128 10.1038/nri2356

[b22] Gaffen S. L. & Liu K. D. Overview of interleukin-2 function, production and clinical applications. Cytokine 28, 109–123 (2004). 15473953 10.1016/j.cyto.2004.06.010

[b23] Boyman O. & Sprent J. The role of interleukin-2 during homeostasis and activation of the immune system. Nat. Rev. Immunol. 12, 180–190 (2012). 22343569 10.1038/nri3156

[b24] Alcami A., Symons J. A., Collins P. D., Williams T. J. & Smith G. L. Blockade of chemokine activity by a soluble chemokine binding protein from vaccinia virus. J. Immunol. 160, 624–633 (1998). 9551896

[b25] Smith C. A. . Poxvirus genomes encode a secreted, soluble protein that preferentially inhibits beta chemokine activity yet lacks sequence homology to known chemokine receptors. Virology 236, 316–327 (1997). 9325239 10.1006/viro.1997.8730

[b26] Graham K. A. . The T1/35kDa family of poxvirus-secreted proteins bind chemokines and modulate leukocyte influx into virus-infected tissues. Virology 229, 12–24 (1997). 9123853 10.1006/viro.1996.8423

[b27] Bahar M. W. . Structure and function of A41, a vaccinia virus chemokine binding protein. PLoS Pathog. 4, e5 (2008). 18208323 10.1371/journal.ppat.0040005 PMC2211551

[b28] Carfi A., Smith C. A., Smolak P. J., McGrew J. & Wiley D. C. Structure of a soluble secreted chemokine inhibitor vCCI (p35) from cowpox virus. Proc. Natl Acad. Sci. USA 96, 12379–12383 (1999). 10535930 10.1073/pnas.96.22.12379 PMC22925

[b29] Zhang L. . Solution structure of the complex between poxvirus-encoded CC chemokine inhibitor vCCI and human MIP-1beta. Proc. Natl Acad. Sci. USA 103, 13985–13990 (2006). 16963564 10.1073/pnas.0602142103 PMC1599900

[b30] Arnold P. L. & Fremont D. H. Structural determinants of chemokine binding by an Ectromelia virus-encoded decoy receptor. J. Virol. 80, 7439–7449 (2006). 16840324 10.1128/JVI.00576-06 PMC1563704

[b31] Counago R. M. . Structures of Orf virus chemokine binding protein in complex with host chemokines reveal clues to broad binding specificity. Structure 23, 1199–1213 (2015). 26095031 10.1016/j.str.2015.04.023

[b32] Hansen G. . The structure of the GM-CSF receptor complex reveals a distinct mode of cytokine receptor activation. Cell 134, 496–507 (2008). 18692472 10.1016/j.cell.2008.05.053

[b33] Broughton S. E. . Conformational changes in the GM-CSF receptor suggest a molecular mechanism for affinity conversion and receptor signaling. Structure 24, 1271–1281 (2016). 27396825 10.1016/j.str.2016.05.017

[b34] Haman A. . Molecular determinants of the granulocyte-macrophage colony-stimulating factor receptor complex assembly. J. Biol. Chem. 274, 34155–34163 (1999). 10567387 10.1074/jbc.274.48.34155

[b35] Rajotte D. . Crucial role of the residue R280 at the F′-G′ loop of the human granulocyte/macrophage colony-stimulating factor receptor alpha chain for ligand recognition. J. Exp. Med. 185, 1939–1950 (1997). 9166423 10.1084/jem.185.11.1939 PMC2196330

[b36] Byun M., Wang X., Pak M., Hansen T. H. & Yokoyama W. M. Cowpox virus exploits the endoplasmic reticulum retention pathway to inhibit MHC class I transport to the cell surface. Cell Host Microbe 2, 306–315 (2007). 18005752 10.1016/j.chom.2007.09.002

[b37] Franzosa E. A. & Xia Y. Structural principles within the human-virus protein-protein interaction network. Proc. Natl Acad. Sci. USA 108, 10538–10543 (2011). 21680884 10.1073/pnas.1101440108 PMC3127880

[b38] Wang X., Rickert M. & Garcia K. C. Structure of the quaternary complex of interleukin-2 with its alpha, beta, and gammac receptors. Science 310, 1159–1163 (2005). 16293754 10.1126/science.1117893

[b39] Levin A. M. . Exploiting a natural conformational switch to engineer an interleukin-2 'superkine'. Nature 484, 529–533 (2012). 22446627 10.1038/nature10975 PMC3338870

[b40] Nelson C. A., Epperson M. L., Singh S., Elliott J. I. & Fremont D. H. Structural conservation and functional diversity of the poxvirus immune evasion (PIE) domain superfamily. Viruses 7, 4878–4898 (2015). 26343707 10.3390/v7092848 PMC4584292

[b41] Elegheert J. . Allosteric competitive inactivation of hematopoietic CSF-1 signaling by the viral decoy receptor BARF1. Nat. Struct. Mol. Biol. 19, 938–947 (2012). 22902366 10.1038/nsmb.2367

[b42] Yang Z., West A. P. & Bjorkman P. J. Crystal structure of TNFalpha complexed with a poxvirus MHC-related TNF binding protein. Nat. Struct. Mol. Biol. 16, 1189–1191 (2009). 19838188 10.1038/nsmb.1683 PMC2819277

[b43] Alejo A. . A chemokine-binding domain in the tumor necrosis factor receptor from variola (smallpox) virus. Proc. Natl Acad. Sci. USA 103, 5995–6000 (2006). 16581912 10.1073/pnas.0510462103 PMC1458686

[b44] Xue X. . Structural basis of chemokine sequestration by CrmD, a poxvirus-encoded tumor necrosis factor receptor. PLoS Pathog. 7, e1002162 (2011). 21829356 10.1371/journal.ppat.1002162 PMC3145792

[b45] McCoy W. H., Wang X., Yokoyama W. M., Hansen T. H. & Fremont D. H. Structural mechanism of ER retrieval of MHC class I by cowpox. PLoS Biol. 10, e1001432 (2012). 23209377 10.1371/journal.pbio.1001432 PMC3507924

[b46] Alexander J. M. . Structural basis of chemokine sequestration by a herpesvirus decoy receptor. Cell 111, 343–356 (2002). 12419245 10.1016/s0092-8674(02)01007-3

[b47] Gileva I. P. . Properties of the recombinant TNF-binding proteins from variola, monkeypox, and cowpox viruses are different. Biochim. Biophys. Acta 1764, 1710–1718 (2006). 17070121 10.1016/j.bbapap.2006.09.006 PMC9628946

[b48] Yang Z., West A. P. Jr. & Bjorkman P. J. Crystal structure of TNFalpha complexed with a poxvirus MHC-related TNF binding protein. Nat. Struct. Mol. Biol. 16, 1189–1191 (2009). 19838188 10.1038/nsmb.1683 PMC2819277

[b49] Aricescu A. R., Lu W. & Jones E. Y. A time- and cost-efficient system for high-level protein production in mammalian cells. Acta Crystallogr. D Biol. Crystallogr. 62, 1243–1250 (2006). 17001101 10.1107/S0907444906029799

[b50] Verstraete K. . Efficient production of bioactive recombinant human Flt3 ligand in *E. coli*. Protein J. 28, 57–65 (2009). 19184382 10.1007/s10930-009-9164-5

[b51] Kabsch W. Xds. Acta Crystallogr. D Biol. Crystallogr. 66, 125–132 (2010). 20124692 10.1107/S0907444909047337 PMC2815665

[b52] McCoy A. J. . Phaser crystallographic software. J. Appl. Crystallogr. 40, 658–674 (2007). 19461840 10.1107/S0021889807021206 PMC2483472

[b53] Rozwarski D. A., Diederichs K., Hecht R., Boone T. & Karplus P. A. Refined crystal structure and mutagenesis of human granulocyte-macrophage colony-stimulating factor. Proteins 26, 304–313 (1996). 8953651 10.1002/(SICI)1097-0134(199611)26:3<304::AID-PROT6>3.0.CO;2-D

[b54] Adams P. D. . PHENIX: a comprehensive Python-based system for macromolecular structure solution. Acta Crystallogr. D Biol. Crystallogr. 66, 213–221 (2010). 20124702 10.1107/S0907444909052925 PMC2815670

[b55] Strong M. . Toward the structural genomics of complexes: crystal structure of a PE/PPE protein complex from *Mycobacterium tuberculosis*. Proc. Natl Acad. Sci. USA 103, 8060–8065 (2006). 16690741 10.1073/pnas.0602606103 PMC1472429

[b56] Blanc E. . Refinement of severely incomplete structures with maximum likelihood in BUSTER-TNT. Acta Crystallogr. D Biol. Crystallogr. 60, 2210–2221 (2004). 15572774 10.1107/S0907444904016427

[b57] Howarth M. & Ting A. Y. Imaging proteins in live mammalian cells with biotin ligase and monovalent streptavidin. Nat. Protoc. 3, 534–545 (2008). 18323822 10.1038/nprot.2008.20 PMC2671200

[b58] Ludtke S. J., Baldwin P. R. & Chiu W. EMAN: semiautomated software for high-resolution single-particle reconstructions. J. Struct. Biol. 128, 82–97 (1999). 10600563 10.1006/jsbi.1999.4174

[b59] Scheres S. H. RELION: implementation of a Bayesian approach to cryo-EM structure determination. J. Struct. Biol. 180, 519–530 (2012). 23000701 10.1016/j.jsb.2012.09.006 PMC3690530

[b60] Cheng Y., Grigorieff N., Penczek P. A. & Walz T. A primer to single-particle cryo-electron microscopy. Cell 161, 438–449 (2015). 25910204 10.1016/j.cell.2015.03.050 PMC4409659

[b61] Penczek P. A. sxviper. *SPARX Wiki* http://sparx-em.org/sparxwiki/sxviper (2014).

[b62] Penczek P. A. sx3dvariability. *SPARX Wiki* http://sparx-em.org/sparxwiki/sx3dvariability (2014).

[b63] Mindell J. A. & Grigorieff N. Accurate determination of local defocus and specimen tilt in electron microscopy. J. Struct. Biol. 142, 334–347 (2003). 12781660 10.1016/s1047-8477(03)00069-8

[b64] Estrozi L. F. & Navaza J. Fast projection matching for cryo-electron microscopy image reconstruction. J. Struct. Biol. 162, 324–334 (2008). 18353677 10.1016/j.jsb.2008.01.014

[b65] Eswar N. . Comparative protein structure modeling using Modeller. Curr. Protoc. Bioinformatics Chapter 5, 6 (2006). 10.1002/0471250953.bi0506s15 PMC4186674 18428767

[b66] Pettersen E. F. . UCSF Chimera--a visualization system for exploratory research and analysis. J. Comput. Chem. 25, 1605–1612 (2004). 15264254 10.1002/jcc.20084

[b67] Konarev P. V., Volkov V. V., Sokolova A. V., Koch M. H. J. & Svergun D. I. PRIMUS : a Windows PC-based system for small-angle scattering data analysis. J. Appl. Crystallogr. 36, 1277–1282 (2003).

[b68] Guinier A. La diffraction des rayons X aux tres petits angles: applications a l'etude de phenomenes ultramicroscopiques. Ann. Phys. 12, 161–237 (1939).

[b69] Rambo R. P. & Tainer J. A. Accurate assessment of mass, models and resolution by small-angle scattering. Nature 496, 477–481 (2013). 23619693 10.1038/nature12070 PMC3714217

[b70] Petoukhov M. V. . New developments in the ATSAS program package for small-angle scattering data analysis. J. Appl. Crystallogr. 45, 342–350 (2012). 25484842 10.1107/S0021889812007662 PMC4233345

[b71] Fischer H., de Oliveira Neto M., Napolitano H. B., Polikarpov I. & Craievich a. F. Determination of the molecular weight of proteins in solution from a single small-angle X-ray scattering measurement on a relative scale. J. Appl. Crystallogr. 43, 101–109 (2009).

[b72] Weinkam P., Pons J. & Sali A. Structure-based model of allostery predicts coupling between distant sites. Proc. Natl Acad. Sci. USA 103, 6 (2012). 10.1073/pnas.1116274109 PMC3324024 22403063

[b73] Schneidman-Duhovny D., Hammel M. & Sali A. FoXS: a web server for rapid computation and fitting of SAXS profiles. Nucleic Acids Res. 38, W540–W544 (2010). 20507903 10.1093/nar/gkq461 PMC2896111

[b74] Dolinsky T. J. . PDB2PQR: expanding and upgrading automated preparation of biomolecular structures for molecular simulations. Nucleic acids Res. 35, W522–W525 (2007). 17488841 10.1093/nar/gkm276 PMC1933214

[b75] Baker N. A., Sept D., Joseph S., Holst M. J. & McCammon J. A. Electrostatics of nanosystems: application to microtubules and the ribosome. Proc. Natl Acad. Sci. USA 98, 10037–10041 (2001). 11517324 10.1073/pnas.181342398 PMC56910

[b76] Armougom F. . Expresso: automatic incorporation of structural information in multiple sequence alignments using 3D-Coffee. Nucleic Acids Res. 34, W604–W608 (2006). 16845081 10.1093/nar/gkl092 PMC1538866

[b77] Robert X. & Gouet P. Deciphering key features in protein structures with the new ENDscript server. Nucleic Acids Res. 42, W320–W324 (2014). 24753421 10.1093/nar/gku316 PMC4086106

[b78] McWilliam H. . Analysis Tool Web Services from the EMBL-EBI. Nucleic Acids Res. 41, W597–W600 (2013). 23671338 10.1093/nar/gkt376 PMC3692137

[b79] Kearse M. . Geneious Basic: an integrated and extendable desktop software platform for the organization and analysis of sequence data. Bioinformatics 28, 1647–1649 (2012). 22543367 10.1093/bioinformatics/bts199 PMC3371832

[b80] Karplus P. A. & Diederichs K. Linking crystallographic model and data quality. Science 336, 1030–1033 (2012). 22628654 10.1126/science.1218231 PMC3457925

