# Supplementary material for: Structural basis of GM-CSF and IL-2 sequestration by the viral decoy receptor GIF
Source: Nat Commun. 2016 Nov 7;7:13228. doi: 10.1038/ncomms13228 (PMC5103067; doi:10.1038/ncomms13228)
Supplement: Supplementary Information — Supplementary Figures 1-6 and Supplementary Tables 1-5. [file ncomms13228-s1.pdf]

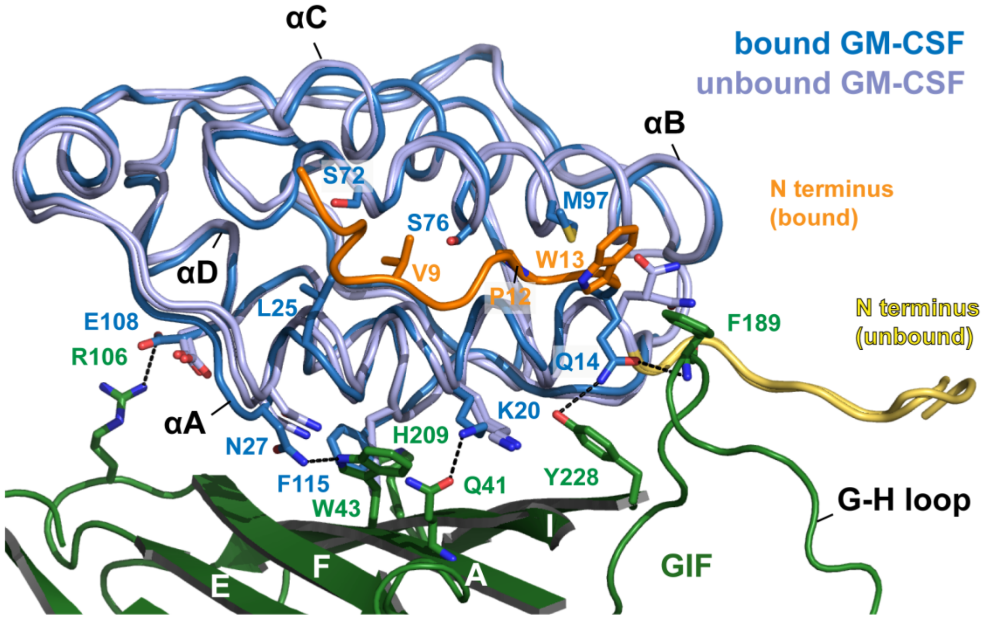

Supplementary figure 1. Comparison of unbound oGM-CSF and oGM-CSF as captured in the GIF:GM-CSF complex. Alignment of two copies of unbound ovine GM-CSF (slate) with bound GM-CSF in the GIF:GM-CSF complex (GIF: green, bound GM-CSF: dark blue). Upon binding, the unstructured N-terminal loop (unbound, yellow) preceding helix αA of GM-CSF is swung back almost 180° (bound, orange) and makes interactions with helix αB of GM-CSF and the G-H loop of GIF. Residues showing large conformational changes are labeled and shown as sticks, polar interactions are shown as dashed lines.

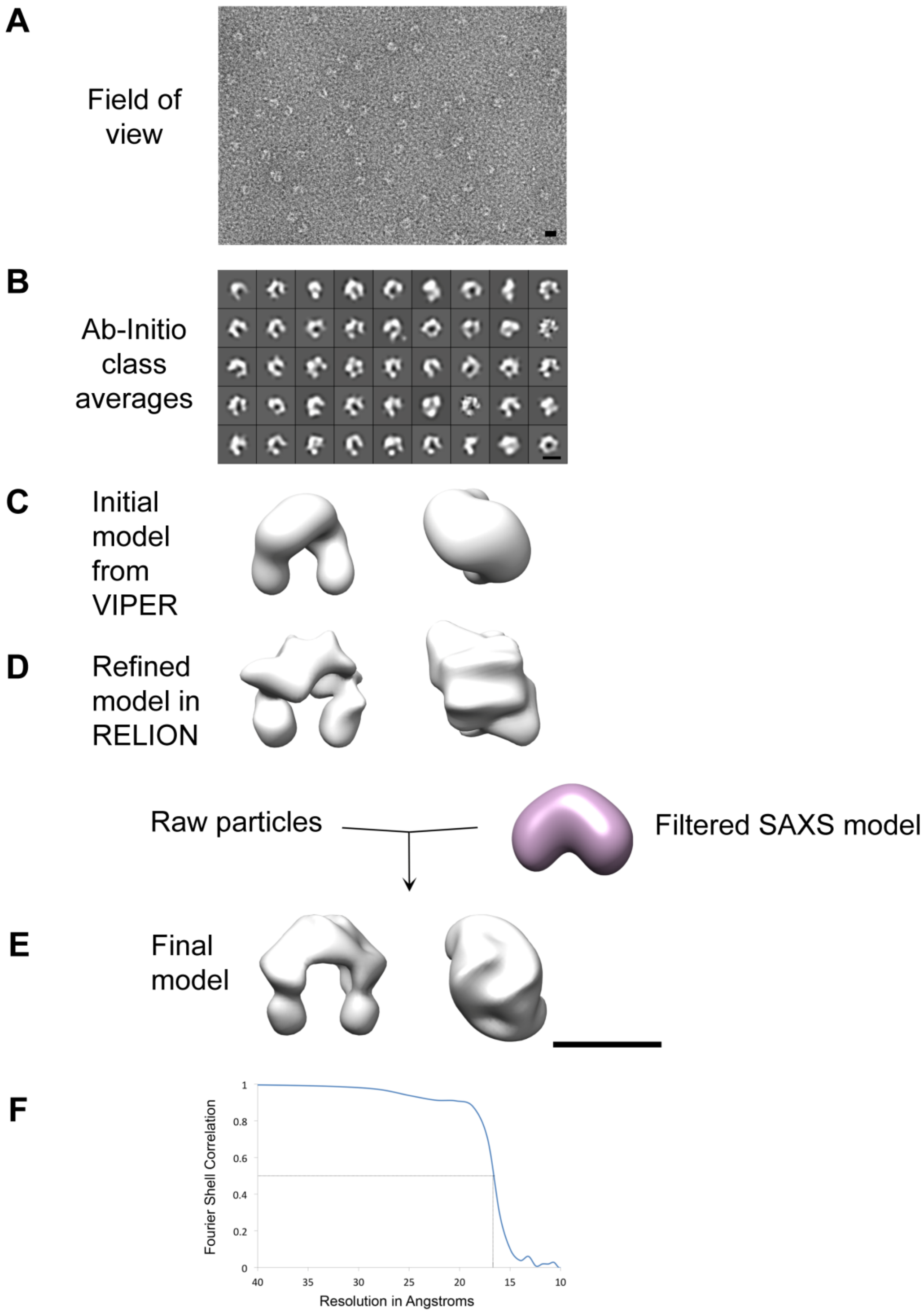

Supplementary figure 2. Rationale and work flow for the analysis of EM data for GIF:IL-2 data. A. Field of view. B. Reference-free class-averages. Three different partly overlapping subsets of these class averages were used to calculate the initial VIPER model. C. The initial VIPER model used for RELION refinement. D. The refined RELION map using the VIPER model. E. The final RELION map obtained after refinement with a SAXS model of GIF:IL2 as a reference model. F. The FSC curve for the final GIF:IL-2 reconstruction.

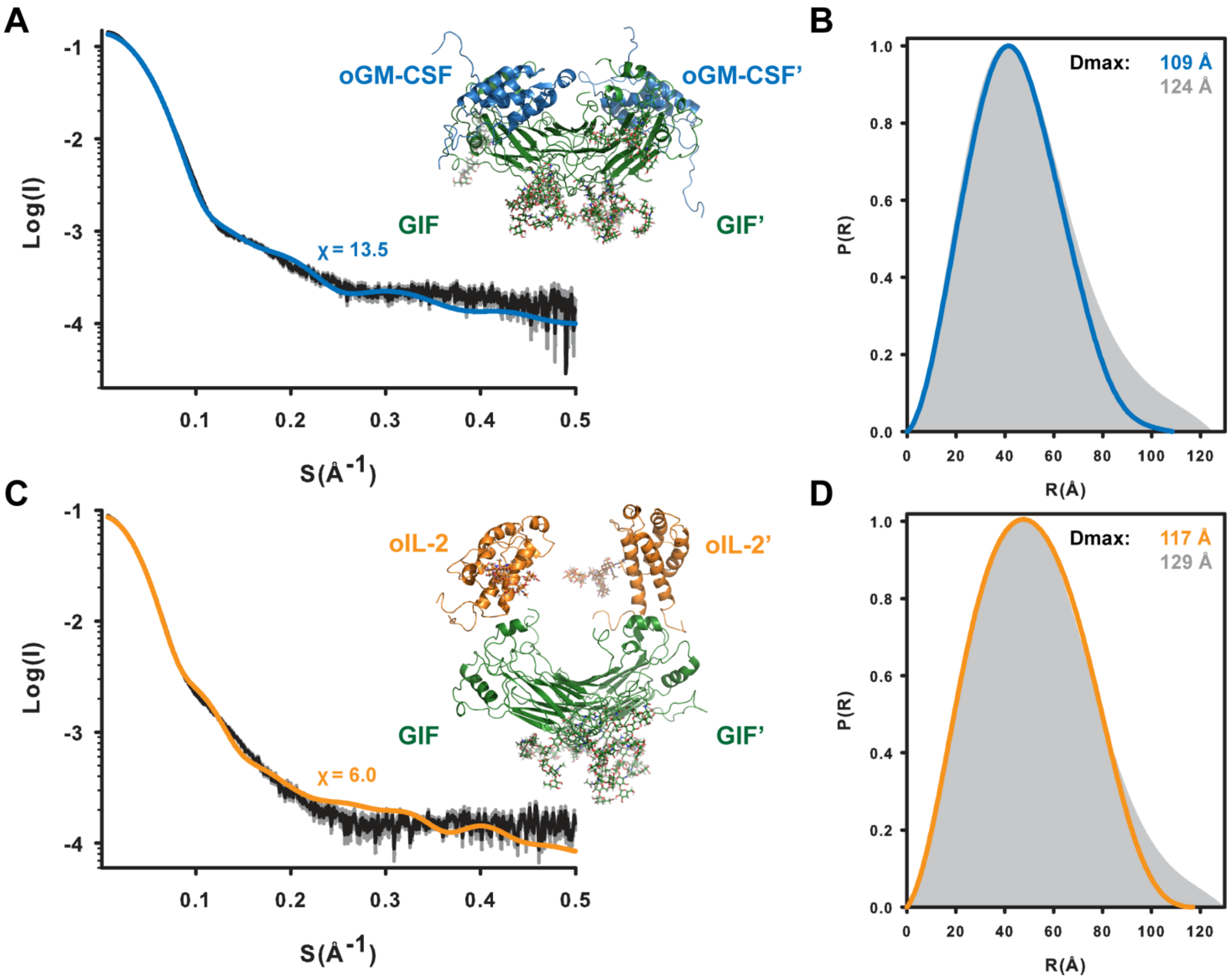

Supplementary figure 3: SAXS analysis of GIF:GM-CSF and GIF:IL-2 complexes. (A & C) Calculated FoXS fits to experimental SAXS data (black curves, A: GIF:GM-CSF and C: GIF:IL-2) of the GIF:GM-CSF crystal structure (A, blue curve) and GIF:IL-2 EM model (C, orange curve) after modeling missing loops and N-linked glycans using Allosmod-FoXS. (B & D) Distance distribution functions and corresponding D_{max} values are shown for the models represented in (A) and (C) alongside the distance distribution function of the experimental data (grey).

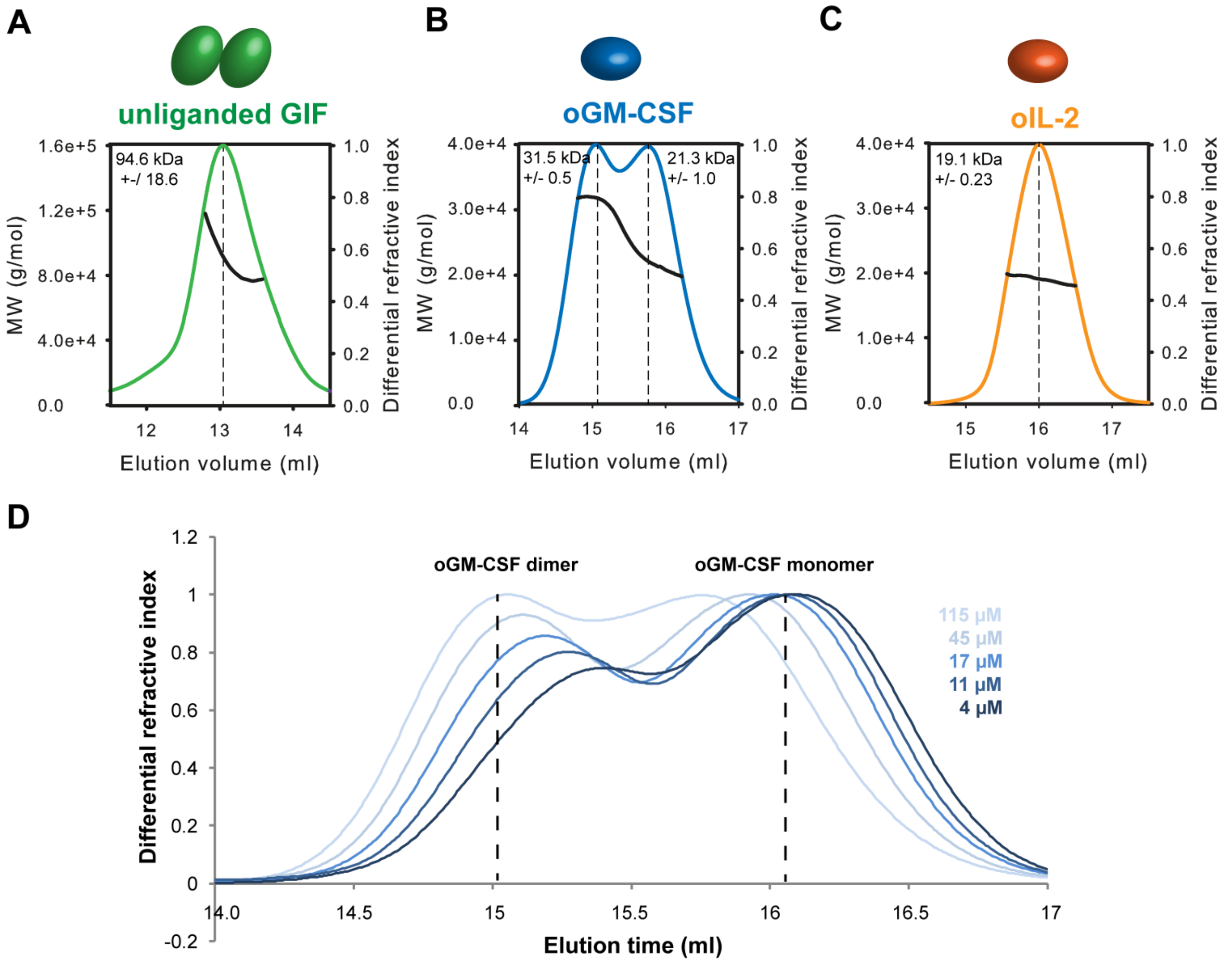

Supplementary figure 4. SEC-MALLS analysis of unliganded GIF (A), oGM-CSF (B) and oIL-2 (C) produced in HEK293T cells. (D) The formation of a dimeric GM-CSF species, eluting at 15 ml, is concentration dependent.

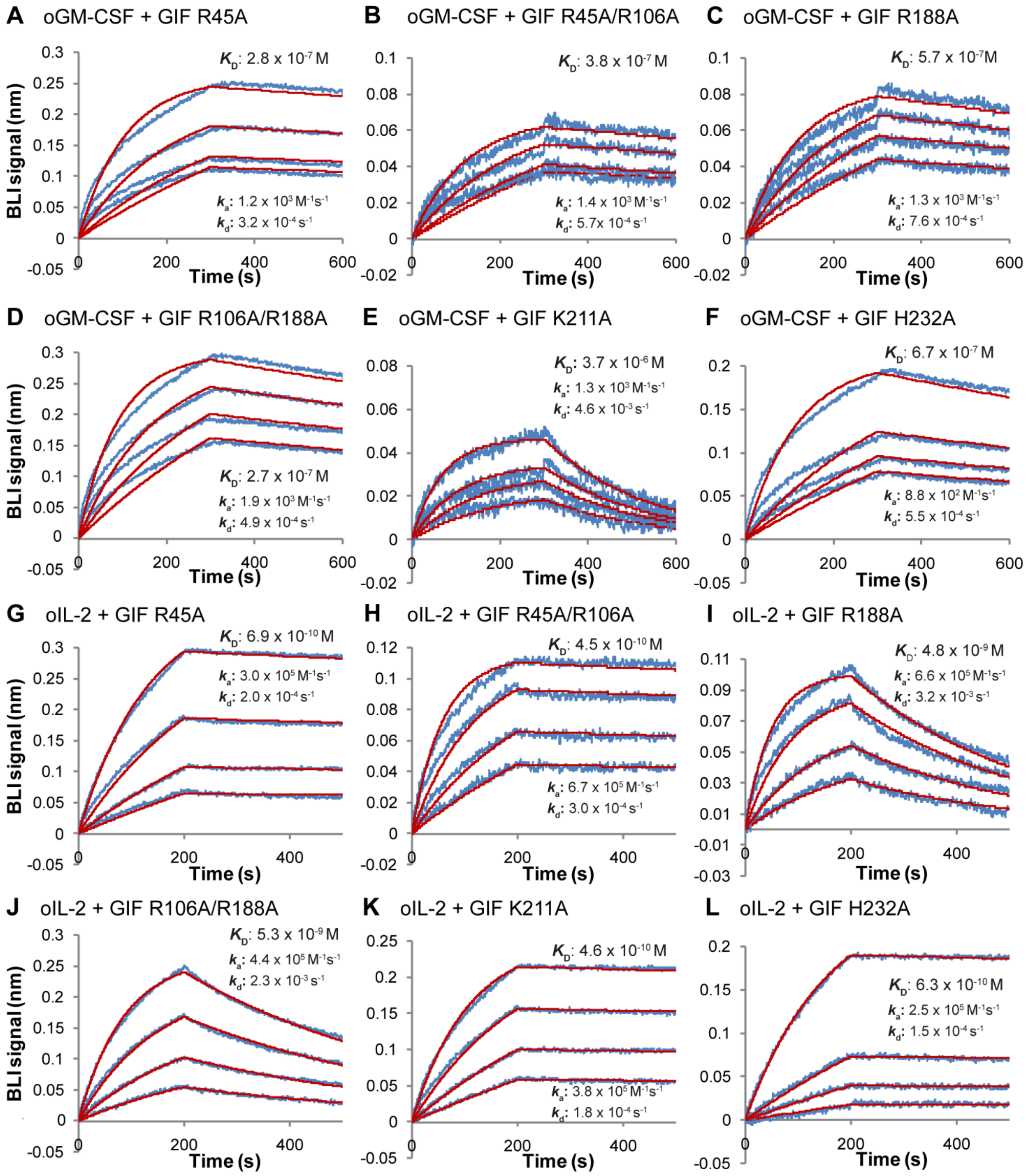

Supplementary figure 5. (A-L) BLI kinetic profiles for the interaction of GIF interface residue mutants (R45A, R45A/R106A, R188A, R106A/R188A, K211A and H232A) with oGM-CSF (A-F) and oIL-2 (G-L). Displayed K_D, k_d and k_a values represent the average of three replicate experiments. The errors on the average K_D, k_d and k_a values are provided in Supplementary Table 5.

A

B

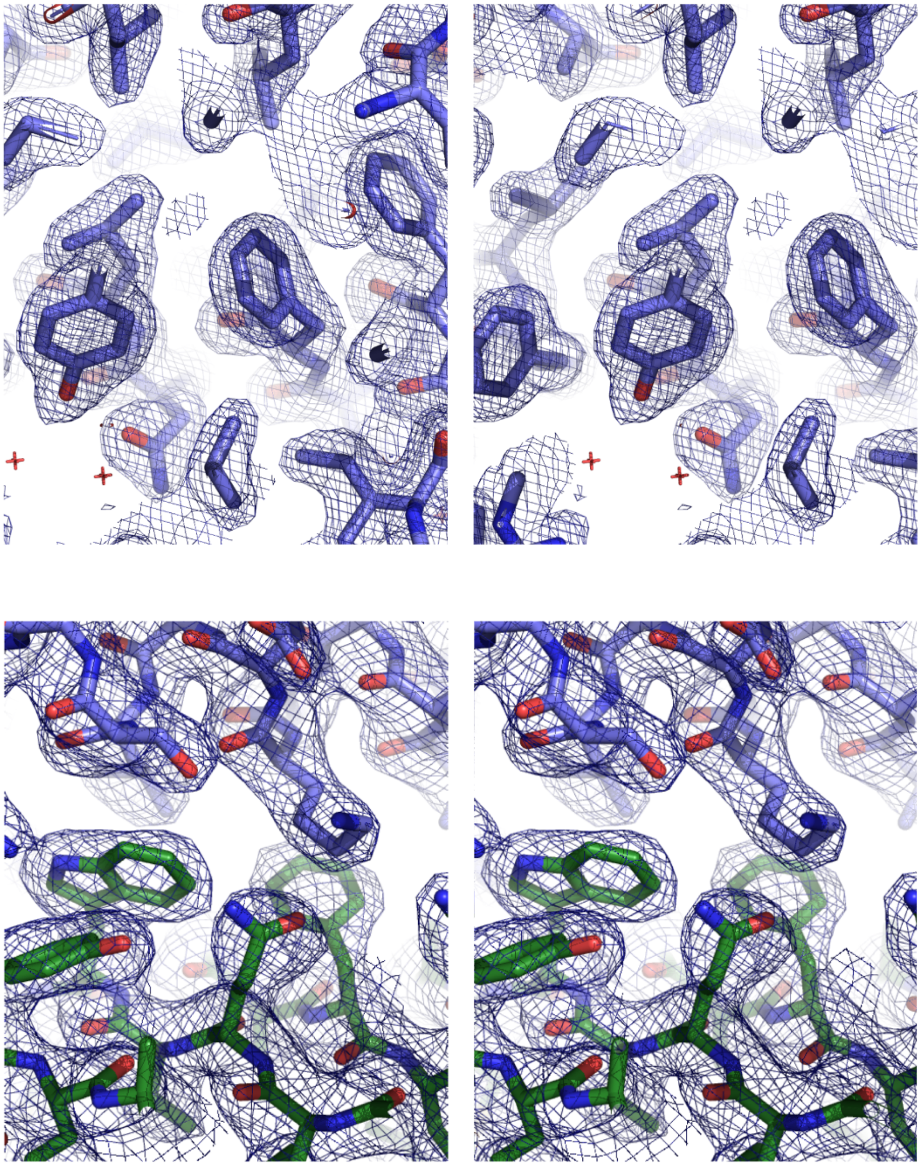

Supplementary figure 6. Stereo-views of electron density maps (2Fo-Fc; α_c) for oGM-CSF (A) and the GIF:oGM-CSF complex (B), contoured at 1.0 σ r.m.s.d. Structures of oGM-CSF (slate blue) and GIF (dark green) are represented as sticks. Panel B shows a zoom of the GM-CSF binding site on GIF, centered around Trp 43 of GIF.

Supplementary Table 1. Observed interactions at the dimer interface between neighboring GIF monomers.

Hydrogen Bonds

GIF	*GIF'*	*Distance (Å)*
Cys59 N	Leu61 O	2.99
Cys59 O	Leu61 N	2.90
Leu61 N	Cys59 O	2.87
Leu61 O	Cys59 N	2.93
Ile63 O	Arg 257 Nη1	2.87
Ser256 O	Ala264 N	3.59
Gly159 N	Gln161 Oε1	3.10
Arg257 Nη1	Ile63 O	2.99
Lys258 N	Gln262 O	2.92
Lys258 Nζ	Gln262 Oε1	3.37
Lys258 O	Gln262 N	2.75
Lys258 O	Gln262 Nε2	3.79
Ser260 N	Ser260 O	3.00
Ser260 O	Ser260 N	3.03
Ser260 Oγ	Ser260 Oγ	3.54
Gln 262 N	Lys258 O	2.86
Gln262 O	Lys 258 N	3.00
Gln262 Oε1	Lys258 Nζ	3.54
Ala264 N	Ser256 O	3.34

van der Waals contacts

GIF	*GIF'*
Ala58	Ala62
Cys59	Ala60
	Met261
Leu61	Ala58
Gln161	Gln161
Ile255	Ala264
Ser256	Ala264
Arg257	Ile63
	Met261
	Thr263
Cys259	Ser260
	Met261
Met261	Tyr102
	Lys258
Gln262	Arg257

*Protein-protein interactions observed in the 2.84 Å structure of the GIF:oGM-CSF complex (PDB ID: 5D28) were analyzed using the PISA server at EBI (http://www.ebi.ac.uk/msd-srv/prot_int/cgi-bin/piserver) and the 'Find Clashes/Contacts' function in Chimera .

Supplementary Table 2. Observed interactions at the GIF:oGM-CSF interface.

Hydrogen Bonds

oGM-CSF	*GIF*	*Distance (Å)*
Asp17 Oδ1	Tyr228 OH	2.17
Asp17 Oδ2	Arg188 Nη2	2.77
Lys20 Nζ	Gln41 Oε1	2.81
Leu23 O	Trp43 Nε1	3.81
Asn27 Oδ1	Trp43 Nε1	2.90
Asp112 Oδ1	Tyr110 OH	2.65
Asp112 Oδ2	Gln108 Nε2	3.26
Leu114 O	Lys211 Nζ	2.54
Phe115 O	Lys211 Nζ	3.01
Ile116 O	His232 Nε2	3.40
Ile117 O	Lys211 Nζ	2.43

Salt Bridges

oGM-CSF	*GIF*	*Distance (Å)*
Asp17 Oδ1	Arg188 Nη2	3.94
Asp17 Oδ2	Arg188 Nη1	3.32
Asp17 Oδ2	Arg188 Nη2	2.77
Glu108 Oε1	Arg106 Nη2	3.82
Asp112 Oδ1	Arg45 Nε	3.25
Asp112 Oδ1	Arg45 Nη1	3.70
Asp112 Oδ1	Arg45 Nη2	3.39
Asp112 Oδ2	Arg45 Nη2	3.22

van der Waals contacts

oGM-CSF	*GIF*	*oGM-CSF*	*GIF*
Thr10	Ala220	Glu108	Tyr110
Trp13	Arg188	Asp112	Arg45
	Phe189	Lys114	Tyr110
Gln14	Phe189	Phe115	Trp43
	Pro191		Met44
Val16	Pro191		Arg45
	Phe213		Tyr110
	Ala230		His209
Ile19	Lys211		Lys211
Lys20	Phe213		His232
	Tyr228	Ile116	Arg45
Leu23	Trp43	Pro118	His232
	Lys211	Phe119	Lys211
Ser24	Trp43		Phe213
Asn27	Trp43		Gly231
	Tyr112		His232

Protein-protein interactions observed in the 2.84 Å structure of the GIF:oGM-CSF complex (PDB ID: 5D28) were analyzed using the PISA server at EBI (http://www.ebi.ac.uk/msd-srv/prot_int/cgi-bin/piserver) and the 'Find Clashes/Contacts' function in Chimera.

Supplementary Table 3. Small-angle X-ray scattering (SAXS) analysis of GIF:oGM-CSF and GIF:oIL-2 complexes.

	GIF:oGM-CSF	GIF:oIL-2
**SASBDB accession code:**	SASDA89	SASDA 99
**Data-collection parameters:**		
Beamline:	SWING, Soleil	SWING, Soleil
Detector:	AVIEX PCCD	AVIEX PCCD
Beam geometry:	0.45 x 0.02 mm^2	0.45 x 0.02 mm^2
Wavelength (Å):	1.0	1.0
q range ($Å^{-1}$):	0.01–0.550	0.01–0.622
Exposure time (s)	1	1
Concentration before injection (mg/ml):	10	8.8
Temperature (K):	293	293
**Structural parameters:**		
I(0) [from P(r)]:	0.15	0.09
Rg (Å) [from P(r)]:	37.34	40.13
I(0) [from Guinier]:	0.15 +- 0.000	0.09 +- 0.000
Rg (Å) [from Guinier]:	37.92 +- 0.120	40.54 +- 0.110
Dmax (Å):	124	129
Porod volume estimate, V_p ($Å^3$) (GNOM):	232454	253425
**Molecular-mass determination:**		
Molecular mass M_r (Da) [from I(0)]:	-	
Molecular mass M_r (Da) [from SaxsMOW]:	144903 (qm: 0.3)	155699 (qm: 0.3)
Molecular mass M_r (Da) [from SCÅTTER]:	110000	100000
Molecular mass Mr (Da) from Porod volume (V_p/1.7)	136737	149074
Calculated M_r (Da) from sequence [2:2]:	89738	92036
Calculated M_r (Da) from sequence [2:2,+N-glycans]:	103690	105988
**Modeling parameters:**		
Modeling of glycosylated complexes	*Allosmod-FoXS*	*Allosmod-FoXS*
Symmetry	None	None
χ (representative model, 2:2 + N-glycans)	13.56	6.00
**Software employed:**		
Primary data reduction:	*FOXTROT*	*FOXTROT*
Data processing:	*PRIMUS-QT, SCÅTTER*	*PRIMUS-QT, SCÅTTER*
Data evaluation:	*PRIMUS-QT, GNOM, SaxsMOW, SCÅTTER*	*PRIMUS-QT, GNOM, SaxsMOW, SCÅTTER*
Computation of model intensities:	*FoXS*	*FoXS*

Supplementary Table 4. Theoretical and measured MW of oGM-CSF, oIL-2, GIF and GIF:GM-CSF/ GIF:IL-2 complexes using multi-angle laser light scattering (MALLS).

	Theoretical MW, no N-glycans (kDa)	Theoretical MW, + N-glycans (kDa)	Measured MW (kDa)
**oGM-CSF monomer**	15.808	17.552 (1 N-glycan)	21.3 +/- 1.0
**oGM-CSF dimer**	31.616	35.104	31.5 +/- 0.5
**oIL-2**	16.957	18.701 (1 N-glycan)	19.0 +/- 0.6
**GIF dimer**	58.122	68.586 (6 N-glycans)	94.6 +/- 18,6
**GIF:oGM-CSF (2:2)**	89.738	103.690 (8 N-glycans)	110.4 +/- 3.0
**GIF:oIL-2 (2:2)**	92.036	105.988 (8 N-glycans)	129.5 +/- 6.9

Supplementary Table 5. Summary of BLI binding studies of wild type and mutant GIF species.

	K_D (M)	k_a(1/Ms)	k_d(1/s)	Fold Affinity Reduction K_D mut/ K_D WT	k_a mut/ k_a WT	k_d mut/ k_d WT
**GIF_WT**						
**oIL-2**	4.7E-10	3.2E+05	1.5E-04			
	+/- 1.0E-10	+/- 5.2E+04	+/- 5.0E-05			
**oGM-CSF**	2.7E-08	2.5E+03	6.3E-05			
	+/- 6.5E-09	+/- 1.9E+03	+/- 3.7E-05			
**GIF_R45A**						
**oIL-2**	6.9E-10	3.0E+05	2.0E-04	1.5	0.9	1.3
	+/- 1.1E-10	+/- 5.1E+04	+/- 6.0E-05			
**oGM-CSF**	2.8E-07	1.2E+03	3.2E-04	10.4	0.5	5.1
	+/- 8.0E-08	+-/ 2.9E+02	+/-1.0E-04			
**GIF_R45A-R106A**						
**oIL-2**	4.5E-10	6.7E+05	3.0E-04	1.0	2.0	2.0
	+/- 1.6E-10	+/- 1.5E+05	+/- 1.1E-04			
**oGM-CSF**	3.8E-07	1.4E+03	5.7E-04	14.1	0.6	9.1
	+/- 8.4E-08	+/- 6.3E+02	+/-3.6E-04			
**GIF_R188A**						
**oIL-2**	4.8E-09	6.6E+05	3.2E-03	10.2	2.1	21.3
	3. 8E-10	+/- 1.1E+05	+/- 2.5E+04			
**oGM-CSF**	5.7E-07	1.3E+03	7.6E-04	21.1	0.5	12.1
	+/- 1.3E-07	+/- 3.8E+02	+/- 3.4E-04			
**GIF_R106A-R188A**						
**oIL-2**	5.3E-09	4.4E+05	2.3E-03	11.3	1.4	15.3
	+/- 9.6E-10	+/- 4.1E+04	+/- 2.3E-04			
**oGM-CSF**	2.7E-07	1.9E+03	4.9E-04	10.0	0.8	7.8
	+/- 8.6E-08	+/- 4.7E+02	+/- 6.2E-05			
**GIF_K211A**						
**oIL-2**	4.6E-10	3.8E+05	1.8E-04	1.0	1.2	1.2
	+/- 1.7E-10	+/-6.7E+04	+/- 9.0E-05			
**oGM-CSF**	3.7E-06	1.3E+03	4.6E-03	137.0	0.5	73.0
	+/- 5.2E-07	+/- 6.4E+01	+/- 5.1E-04			
**GIF_H232A**						
**oIL-2**	6.3E-10	2.5E+05	1.5E-04	1.3	0.8	1.0
	+/- 1.2E-10	+/- 2.7E+04	+/- 2.7E-05			
**oGM-CSF**	6.7E-07	8.8E+02	5.5E-04	24.8	0.4	8.7
	+/- 2.4E-07	+/- 2.3E+02	+/- 1.0E-04			
